# Pervaporation as a Successful Tool in the Treatment of Industrial Liquid Mixtures

**DOI:** 10.3390/polym14081604

**Published:** 2022-04-14

**Authors:** Kadavil Subhash Lakshmy, Devika Lal, Anandu Nair, Allan Babu, Haritha Das, Neethu Govind, Mariia Dmitrenko, Anna Kuzminova, Aleksandra Korniak, Anastasia Penkova, Abhimanyu Tharayil, Sabu Thomas

**Affiliations:** 1 School of Energy Materials, Mahatma Gandhi University, Kottayam 686560, Kerala, India; lakshmyks19@gmail.com (K.S.L.); devikasatheesh2505@gmail.com (D.L.); anandunairm@gmail.com (A.N.); allanbb3@gmail.com (A.B.); haritha.r.das1212@gmail.com (H.D.); neethuvg97@gmail.com (N.G.); sabupolymer@yahoo.com (S.T.); 2 St. Petersburg State University, 7/9 Universitetskaya nab., 199034 St. Petersburg, Russia; m.dmitrienko@spbu.ru (M.D.); a.kuzminova@spbu.ru (A.K.); kornyaksandra@gmail.com (A.K.)

**Keywords:** pervaporation, wastewater, volatile organic compounds

## Abstract

Pervaporation is one of the most active topics in membrane research, and it has time and again proven to be an essential component for chemical separation. It has been employed in the removal of impurities from raw materials, separation of products and by-products after reaction, and separation of pollutants from water. Given the global problem of water pollution, this approach is efficient in removing hazardous substances from water bodies. Conventional processes are based on thermodynamic equilibria involving a phase transition such as distillation and liquid–liquid extraction. These techniques have a relatively low efficacy and nowadays they are not recommended because it is not sustainable in terms of energy consumption and/or waste generation. Pervaporation emerged in the 1980s and is now becoming a popular membrane separation technology because of its intrinsic features such as low energy requirements, cheap separation costs, and good quality product output. The focus of this review is on current developments in pervaporation, mass transport in membranes, material selection, fabrication and characterization techniques, and applications of various membranes in the separation of chemicals from water.

## 1. Introduction

Trichloroethylene (TCE), benzene, toluene, carbon tetrachloride, trichloroethane, and other volatile organic compounds (VOCs) are regularly discovered in contaminated ground water and soil from various industrial and commercial locations. Some of these VOCs have the potential to cause cancer and pose a threat to all living organisms [1]. VOCs can be harmful to the ecology in a multitude of ways due to their volatile nature. Because of their widespread use as cleaners and degreasers, chlorinated hydrocarbons (TCE, perchloroethylene (PCE), and 1,2 dichloroethylene (l,2-DCE)) are common groundwater pollutants. Pervaporation is a membrane-based separation method for binary or multi-component mixtures. The separation of the mixtures is accomplished through the use of a membrane known as pervaporation membrane [2]. These non-porous membranes, which are composed of polymeric or ceramic materials, have variable permeability to different components, enabling the necessary separation of components. The fundamental advantage of pervaporation over distillation is that the separation is not relied on thermodynamic equilibrium between the vapor and liquid phases. This means that the concentration of permeate is not defined by the vapor-liquid equilibrium (VLE) but by the permeability of the compounds through the membrane, which depends on their solubility and diffusion rate in the membrane. There are cases when the pervaporation diagram (the dependence of the permeate composition on the composition of the feed) coincides with the VLE phase diagram. In this case, it is impossible to talk about the effectiveness of the use of membranes for the pervaporation process. The distillation of mixtures with an azeotropic composition or with components with low relative volatility or close-boiling mixtures is also energetically expensive, and auxiliary substances are usually required [3]. However, despite the pervaporation advantages (continuity, low energy costs, ease of combination with other technological processes, mild technological conditions, scalability, absence of necessity of inclusion of additional substances-additives), it has not found wide industrial application at present for a number of reasons: for a specific separation task, a certain membrane material must be selected, which in the process of mass transfer of separated substances and phase transitions will stable and save its transport properties (despite swelling and concentration polarization). It should be noted that the most effective use of pervaporation is the application in combined (hybrid) processes to solve not only technological problems, but also environmental problems associated with environmental pollution.

The feed solution is kept at a specified temperature and pressure during this process such that the temperature is lower than the boiling point and the pressure is higher than the bubble point of the feed, to ensure a liquid phase throughout the operation. The separation requires a non-porous composite (supported) membrane since the membrane is what gives the process its selectivity. This indicates that the membrane is the most important factor in the separation, and the efficacy of pervaporation in separating a given compound from a mixture is determined by the membrane’s selectivity [4], which depends on membrane material. There are several types of pervaporation depending on driving force applied for the separation: vacuum pervaporation (pressure difference), thermo-pervaporation (temperature difference) and pervaporation with sweeping gas. Vacuum pervaporation is the most applied nowadays for the separation [5]. In this mode the permeate side is generally kept under vacuum to ensure a large driving power. Permeate is condensed and collected in a liquid phase and the retentate is concentrated in the less permeant species. The difference in partial pressures of the components on both sides of the membrane provides the driving force for separation. Various transport rates of molecules through the membrane as a result of different solubilities and diffusivities of the components provide separation selectivity [6].

Pervaporation can be classified as hydrophilic pervaporation, hydrophobic pervaporation, or organic pervaporation, depending on the type of the solution to be treated. Hydrophilic pervaporation, in particular, necessitates the use of hydrophilic membrane materials to promote water molecule dispersion and penetration across the membrane [7]. The hydrophobic moiety of the membranes aids in the separation of nonpolar organic molecules from water in hydrophobic pervaporation [8]. Finally, depending on the kind of separation (polar/nonpolar, polar/polar, or nonpolar/nonpolar), both hydrophilic and hydrophobic membrane materials can be employed in organic–organic pervaporation.

The history of pervaporation is closely related to researches of membrane materials and the mass transfer through dense “barriers” [9]. Evaporation of a liquid mixture through a dense membrane condensing to the vapor in the downstream side at a low pressure was firstly discovered by Kahlenberg in 1906 with mixtures of hydrocarbons and alcohol [10]. Then the term pervaporation was firstly identified in 1917 by P.A. Kober and called a perstillation [11]. The formation of this term was due to a combination of “permselective” and “evaporation” words since the feed components in a liquid phase permeate selectively through the membrane and are collected in a vapor phase [12]. Further, in the 1950s and 1960s, the researches were actively carried out on pervaporation separation using dense membranes based on polymers [13,14,15]. From the mid-1960s, Professors J. Néel and P. Aptel (University of Toulouse) made a significant contribution to the investigation of the pervaporation mechanism and the evaluation of its economic efficiency [16,17]. It was demonstrated that the separation efficiency significantly depends on the substance affinity with the membrane material [18], and in the combination of pervaporation with distillation for the separation of azeotropic mixtures it was most expedient to apply pervaporation to extract components with a lower concentration in the feed [16,19,20].

The industrial implementation of the pervaporation began in 1982 by Gesellschaft für Trenntechnik (GFT, Hameln, Germany) manufactured the first industrial pervaporation supported membrane with thin selective layer based on cross-linked polyvinyl alcohol (PVA) deposited on a porous polyacrylonitrile (PAN) substrate [21]. In 1983 the first industrial pervaporation plant for ethanol dehydration was introduced in Brazil by GFT [22]. Thus, since 1982, these innovations have intensified research of this process, and the number of publications and patents devoted to this has begun to increase rapidly [23,24]. To date, most studies of pervaporation is aimed at finding novel membrane materials, as well as investigation of various modification methods to vary and obtained tailored membrane characteristics.

In 2000 the world market for pervaporation was over 10 million Euro. Various types of pervaporation modules are commercially available, such as plate frames, spiral modules, and inorganic multichannel tubes [9]. Nowadays, pervaporation equipment and membranes are manufactured by companies: Pervatech (Rijssen, The Netherlands), Compact Membrane Systems (Newport, DE, USA), BUSS-SMS-Canzler (Butzbach, Germany), Mitsui Zosen Machinery & Service Inc. and Ube Industries Ltd. (Tokyo, Japan), Energy research Centre of the Netherlands (ECN) (Petten, The Netherlands), Vladipor Ltd. (Vladimir, Russia), and so forth. One of the leading manufacturers of pervaporation membranes, modules and installations on the world market is GFT Membrane Systems GmbH (now owned by Sulzer Chemtech GmbH, headquartered since 1834 in Winterthur, Switzerland). Already by 2010, Sulzer Chemtech GmbH has installed more than 200 pervaporation installations, most of them for the dehydration of solvents, alcohols in the pharmaceutical and chemical industries [25]. However, research on the creation and improvement of the properties of membranes is currently very relevant due to the tightening of environmental requirements and the improvement of the efficiency of industrial pervaporation.

Pervaporation techniques are of paramount relevance in the present scenario [26], since there is a problem of lack of drinking water in the world. One of the most reliable water resources available is wastewater, which increases rapidly with population growth, industrial and agricultural activity. Improving wastewater treatment processes for the separation of organic and inorganic pollutants, suspended particles, salts, heavy metals, nutrients, and so forth is necessary to reuse treated wastewater. Membrane technology has become the preferred choice for this. The most widely used membrane processes in wastewater treatment, from pre-treatment to post-treatment, are pressure-driven membrane processes—microfiltration (MF), ultrafiltration (UF), nanofiltration (NO) and reverse osmosis (RO) [27,28]. The main for these processes is the pressure requirements and the pore size of the membrane. However, pervaporation is more promising compared to these methods for the separation of components with close molecular sizes [27]. It is actively used for dehydration of various solvents [29], what cannot be solved using the above methods, for micro irrigation of plants from wastewater [30], to remove organic solvents (benzene, toluene, naphtha, butane, ethyl ether, etc.) from dilute aqueous streams [31,32], aqueous VOCs (ethyl acetate, diethyl ether, acetonitrile) [33], and so forth. In addition to being able to separate liquid mixtures of low molecular weight substances, where traditional separation processes are limited, pervaporation is known as an energy-saving and environmentally friendly technology [27].

Secondly, the process of pervaporation has an upper hand in the separation of compounds that are arduous to segregate by several other techniques such as extraction, absorption, adsorption, distillation, and so forth. For instance, numerous strategies were used to separate lactic acid from fermentation broth. In a recent study by Li C. et al. [34] the method involved the separation technologies based on phase transition (traditional precipitation method, solvent extraction, adsorption and molecular distillation), membrane separation (pervaporation) and esterification. Apparently, it was difficult to achieve a notable separation by any of the mentioned techniques singly; a combination of esterification with pervaporation showed a remarkable performance. Methods such as electrodialysis (ED) and microfiltration (MF) are comparable, and sometimes outperform pervaporation in terms of performance. Gally C.R. et al. [35] recently applied ED for the tertiary treatment of effluents. During a discontinuous treatment over a year, they found that ED was almost 100% efficient according to Brazilian standards and performance. They observed a high amount of ion extraction and a cutback in the electrical conductivity, justifying the usage of ED for separation strategies. However, reusage of the ED membranes would result in a drop in the limiting current density of the effluent, which may be due to the aggregate accumulation from the foulant components from the wastewater.

The removal of heavy metal ions from wastewater is also an important issue as they are highly toxic even at low concentrations and cannot be biodegraded [36]. In the past few decades, processes such as adsorption, biological treatment, advanced oxidation processes, electrodeposition and membrane separation have been used for this purpose [37]. Caprarescu S. et al. [38] emphasized the study of polymeric membrane performance with and without SiO_2_ for the effective removal of Zn ions from wastewater. They used an advanced versatile system for ED. The results proved that the fabricated polymeric membrane was efficient at separating metallic ions from wastewater, and promising to be used for water desalination, metallic ion separation and the treatment of effluents from sewage. In another study, Caprarescu S. et al. [39] fabricated silver and chitosan enriched biopolymer membranes to separate metal ions. The synthesized membrane had high capability to separate iron ions from wastewaters due to the electrical characteristics. These membranes could even be used in fuel cells, membrane techniques such as nanofiltration, ultrafiltration, microfiltration, reverse osmosis, and so forth. Though, there are several separation techniques including electrodialysis, ultrafiltration, microfiltration and many more, pervaporation technique has many merits, including an excellent capability to separate azeotropic mixtures, recover some components from the mixtures, and so forth. [40].

The objective of this work is to review the latest advances in various manufacturing concepts of various pervaporation membranes, membrane materials fabrication techniques and characterization methods. The application of these membranes and its mechanism of operation and mass transport in the separation of various liquid mixtures for its purification and further usage is also stressed.

## 2. Mass Transport Mechanisms within Pervaporation Membranes

**Figure 1:**
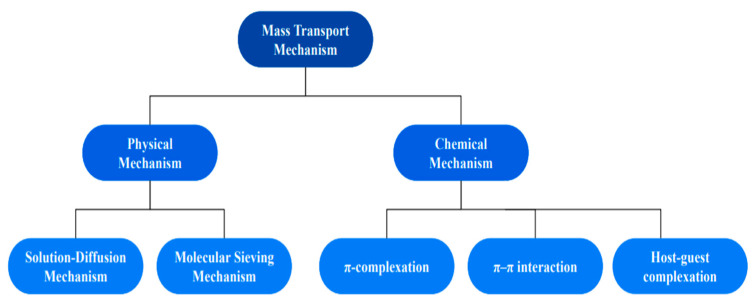
Classification of mechanisms of mass transport.

With the rapid development of new membrane materials, there shows a significant improvement in the performance of pervaporation. In order to design and fabricate membranes with higher performance to meet the technological and economical requirements of industrial application, fundamental understanding of the mass transport mechanism is crucial. Classification of mechanisms of mass transport is shown in Figure 1.

They are classified into physical and chemical mechanisms on the basis of the interactions between permeate molecules and membranes. Physical mechanism is further classified into solution-diffusion and molecular sieving mechanisms whereas chemical mechanism includes facilitated transport mechanism. Chemical mechanism is mainly based on non-specific interactions such as Van der Waals force of interactions whereas chemical mechanism is primarily based on reversible chemical interactions such as π complexation, π–π interaction and host-guest complexation, which involve the formation of chemical bonds within the limit 10–15 kJ/mol of energy [41]. In the physical mechanism of mass transport, separations are realized without the formation of the chemical bonds due to the non-specific interactions and/or steric effect between permeate molecules and membranes. It occurs for most feed molecules in contact with a membrane surface under non-specific interactions such as Van der Waals interactions, weak hydrogen and hydrophobic bonding [42]. 

**Figure 2:**
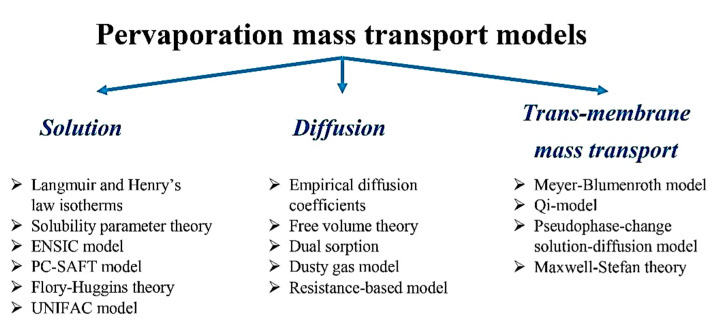
Models used for mass transfer in pervaporation.

The mass transport mechanism within pervaporation membranes can be analyzed with the help of both thermodynamic and kinetic perspectives. Solubility is a thermodynamic parameter, which gives the amount of component sorbed on the surface of a membrane, whereas diffusivity is a kinetic parameter depending on the shape and size of penetrant. The solution-diffusion mechanism is one of the most commonly used mechanisms to describe mass transport through pervaporation non-porous membranes. According to this mechanism, the process of pervaporation is carried out in three steps, which include: (i) solution/sorption of the components from the liquid mixture into the membrane at the upstream side; (ii) diffusion of the components through the membrane; (iii) desorption/evaporation of the permeate to the vapor phase at the downstream side [43]. Solubility selectivity is primarily relevant to the relative condensability of permeates and the relative affinity between permeate molecules and the membrane material [44], while diffusivity selectivity is controlled by the differences in molecular weight, size, and shape of the permeate molecules, as well as the fractional free volume (FFV) of the membrane matrix. The simulation of mass transport mechanisms in the pervaporation process offers quantitative insights into the solution-diffusion mechanism and different models have been reported. Some models are meant only for either the solution or the diffusion step, whereas some models are for the overall trans-membrane mass transport (Figure 2). They can be broadly classified into empirical, semi-empirical, or theoretical models [41]. Concerning the “solution” step of solution-diffusion mechanism, such models are applied for binary mixture separation as Langmuir and Henry’s law isotherms [45], Solubility parameter theory [46], ENSIC model [47], PC-SAFT model [48], Flory-Huggins theory [49], and for the multicomponent mixture separation - Flory-Huggins theory and UNIFAC model [50]. Concerning the “diffusion” step, such models are applied for binary mixture separation as Free volume theory [51], Dual sorption [52], Resistance-based model [53], and for the multicomponent mixture separation - Empirical diffusion coefficients [54] and Dusty gas model [55]. The “trans-membrane mass transport” models applied for binary mixture separation are the Meyer–Blumenroth model [54], Maxwell–Stefan theory [56], Qi-model [54], and for the multicomponent mixture separation - the Pseudophase-change solution-diffusion model [57]. It should be noted that almost all mentioned models are used when pervaporation separation is carried out by polymeric membranes with the exception of Maxwell–Stefan theory that can be used for polymeric and inorganic membranes; the Dusty gas model is used for inorganic membranes and the Resistance-based model is applied for mixed matrix membranes. 

At present, a large amount of pervaporation literature concerning proposals for concentration dependence approaches the diffusion step through Fick’s first law with a concentration dependent diffusivity. According to Fick’s first law, there exists a linear dependence between the diffusion flux of species, its average mixture velocity and composition gradient. For a binary mixture consisting of water (*i*) and an organic solvent (*j*), the partial water flux through a non-porous membrane is given in Equation (1) [58]:

(1) $$J_{i}=\rho_{m}D_{i}\left(w_{im},T\right)\frac{dw_{im}}{dx},$$

where $w_{im}\;$ is the water weight fraction, $\rho_{m}\;$ is the membrane density (kg/m^3^), *x* is the distance from feed/membrane interface (m) and the water diffusivity, $D_{i},\;$ is assumed to be concentration- and temperature-dependent through the relationship in Equation (2) [58]:

(2) $$D_{i}\left(w_{im},\;T\right)=D_{0,i}\left(T\right)w_{im}^{n\left(T\right)},$$

where *n* is the power of the water mass fraction in the membrane, $D_{0,i}$ is the diffusion coefficient at infinite dilution, and these parameters are dependent on the temperature (*T*).

However, the Fick’s binary diffusion equation does not always suit membranes with two or more components. In such cases of a multicomponent system with solute, solvent and membrane, the Maxwell–Stefan equations are found suitable for the transport of two components through the membrane material. For a ternary mixture of solute, solvent and membrane as components 1, 2 and 3, respectively, the transport equation for component 1 is based on the driving force of component 1, and the friction of this component with the membrane and with component 2 [59]. Some models with a liquid-vapor surface inside the membrane follow a transport theory, which is a further development of pore flow mechanism and considered a combination of liquid and vapor transport. A framework of non-equilibrium thermodynamics (NET) is used here for heat and mass transport equations for pervaporation separation of binary mixtures. This theory was first applied to the description of coupled fluxes through membranes, where the system was divided into three phases: (i) the feed–membrane surface, where transport is driven by differences in chemical and temperature potentials, (ii) the membrane and (iii) membrane-permeate surface, where transport is driven by gradients in chemical and temperature potentials. Kuhn et al. [60] investigated the coupled mass and heat transport in the pervaporation of pure water in a zeolite type membrane using the framework of NET. It was found that there existed coupling effects between the heat and mass transport, and the heat flux resulted in an extra driving force for mass transport, reducing the activity over the membrane, but the mass transfer across the interfaces was determined by the connection with the heat flux [60]. In addition to coupled effects, recent works have also addressed other separation processes such as ultrafiltration, nanofiltration, osmosis and reverse osmosis. In a study conducted by Toikka et al. [61], the approach of NET was applied to the pervaporation of water-organic binary mixtures. They discussed about the common VLE models for the calculation of thermodynamic properties of feed solutions. The trans-membrane fluxes in a case of isothermal non-equilibrium process in binary system could also be presented as functions of chemical potential by basic phenomenological equations. In contrast to various approaches such as solution–diffusion models, the NET approach gives clear interpretation of data with lesser complexity and, thus, deemed good for pervaporation process involving multicomponent system.

Another type of physical mechanism is molecular sieving, which is found able to supplement the solution-diffusion mechanism with a trade-off effect between selectivity and permeability. This means that a more permeable material shows less selectivity in most cases of fabrication of membrane materials in pervaporation. According to the molecular sieving mechanism, when the pore size of the membrane falls between the molecular sizes of two components [53], the membrane will reject the large component and allow the small component only to pass through. In short, the membrane in sieving mechanism is promising to exhibit cut off separations. The permeability (*Pi*) for the transport of component *i* is both a function of solubility coefficient (*Si*, cm^3^∙(STP)∙cm^−3^∙cm∙Hg^−1^) and diffusivity coefficient (*Di*, cm^2^∙s^−1^) as shown in the equation (3) [41]:

(3) $$P_{i}=S_{i}\times D_{i}$$

**Figure 3:**
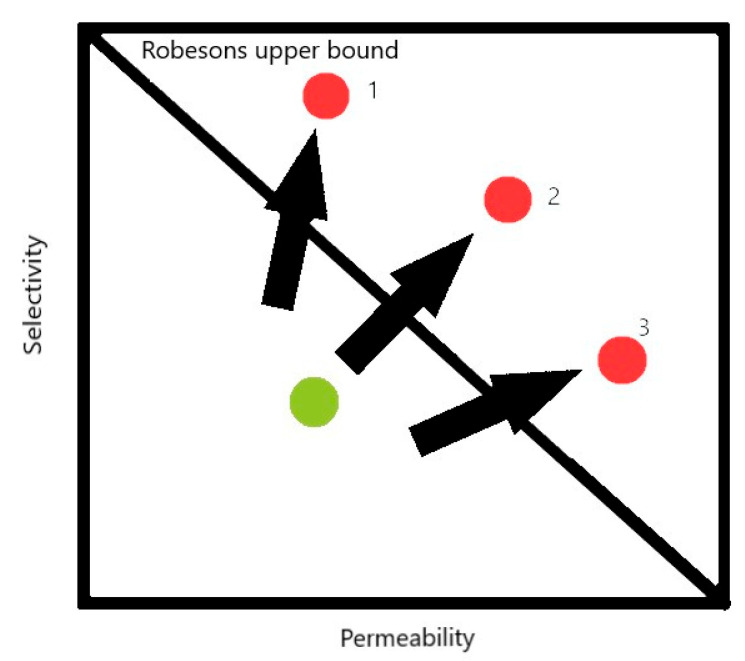
Three cases of possible changes of MMMs transport parameters depending on the modifier.

In 1991 the first empirical trade-off between permeability and molecular selectivity was codified for gas separation membranes by Robeson as an “upper bound” with the help of a double log plot of selectivity vs. permeability [62]. The molecular sieving materials such as zeolites and carbon molecular sieves (CMS) are found to have values above the upper bound polymeric trade-off curve. In a recent work, Zimmerman et al. explored and predicted the potential of mixed matrix membranes (MMMs) with its gas separation performance beyond Robeson’s upper bound, highlighting the need for a hybrid approach to membrane materials development considering deficiencies in both the polymeric and purely molecular sieving media [63]. They designed the MMMs so as to overcome the upper bound, in which the compatibility between filler and polymer, filler particle size and shape, and homogeneous filler distribution were primarily considered. With their experiments, they could draw the facts that molecular sieving fillers such as CMS and zeolites often resulted in an increase in selectivity, but decrease permeability as shown in case 1 in Figure 3 [64]. In case 2 in Figure 3, molecular sieving fillers with nano size or nanosheet shapes such as MOFs nanocrystals or 2D nanosheets help in improving both permeability and selectivity, and case 3 in Figure 3 shows that the fillers with homogeneously dispersed interfacial voids can result in increased permeability and decreased selectivity. For the pervaporation membranes, there are still no special equations and curves showing the trade-off bound for different mixtures such as the Robbeson upper bound for gas separation. However, the comparison of membrane transport properties in the pervaporation separation of the exact mixture is usually presented in terms of the dependence of permeability coefficients and selectivity values calculated from experimental data (permeation flux, separation factor, thickness, etc.) collected from studies [65]. The use of these parameters is due to the fact that they are less sensitive to the variation of operating conditions and membrane thickness than permeation flux and separation factor [65], and related to the intrinsic properties of the separation membranes [66]. 

Considering that there is a strong interaction between the pores and the molecular wall in the transport of liquid molecules, the decisive factor in the molecular sieve is mainly the energy of the interaction of the permeable membrane, which depends on the size of the pores and permeate molecules. Since the highlighted pore measurements for atomic strainers as well as the size contrasts of normal pervade sets are of angstrom scale, exact control of film pore size is exceptionally difficult [67].

In pervaporation, when the bond energy lies within the range of 10–15 kJ/mol [41], the bond is weak enough to be broken by using simple operations. The bonds formed by the chemical interactions are stronger than those by Van der Waals interaction alone, and, thus, possible to achieve high selectivity as well as high capacity for the component to get bound. The chemical interaction is “reversible”, which means the bond is weak enough to be broken by using simple operations such as decreasing the pressure. Thus, the carrier acts as a shuttle to selectively transport of one component from the feed to the permeate side of the membrane. The concentration of target molecules around facilitated transport carriers fluctuates instantaneously due to continuous reversible chemical reactions, which generate high chemical potential gradients and high separation efficiency. Such reversible chemical reactions typically include π-complexation, π–π interaction, and host-guest complexation in pervaporation applications.

In chemical mechanisms, chemical reactions are involved which includes transfer or sharing of electrons between permeate molecules and membrane materials. Chemical bond formation is essential for a chemical enhancement to the physical mechanism. This is known as facilitated transport or carrier mediated transport. Here the transport of components across the membrane is carried out with the help of carriers, which acts as a shuttle for selective transportation of components from the feed to the product side. A high chemical potential gradient as well as a high separation efficiency is generated due to continuous reversible chemical reactions. These facilitated transport mechanisms also based on reversible chemical reactions include π-complexation, π–π interaction, and host-guest complexation.

Most of the π-complexation carriers belong to d-block which include transition metal ions such as Cu^+^, Ag^+^, Cu^2+^, Ni^2+^, Ti^2+^, Pb^2+^, Mn^2+^, Co^2+^, and Cr^3+^ [68]. The primary interactions of π-complexation is carried out by cation-π and π-d interactions. It occurs when the π-orbital of target molecule donates electron charge to the vacant *s* orbital of the metals, known as *s donation*, and, simultaneously, back-donates electron charges from the *d* orbitals of the metals to π* orbital of target molecule, or *p backdonation* [69]. There are several factors upon which the bonding intensity of π-complexation between metallic carriers and target molecules depends on. This includes: (i) emptiness of the outer-shell s-orbital of the cation; (ii) the amount of d-orbital electrons of the cation and the ease with which they can be donated to the target molecule [70]. Analyzing different theoretical calculations and experimental results, π-complexation strengths follow the order Cu^+^ > Ag^+^ > Cu^2+^ > Ni^2+^ > Pd^2+^ [69].

Another type of facilitated transport mechanism is π–π interaction, which are non-covalent intermolecular interactions between aromatic rings. The aromatic components involved attract each other via π–π interactions and in addition, contribute to self-assembly and molecular recognition processes, which requires an average energy of about 2 kJ/mol for a typical π-stacking interaction [71]. However, macromolecules such as graphite, graphene, carbon nanotubes (CNTs), C_60_ and molybdenum disulfide (MoS_2_), have large aromatic clusters thereby strengthening π–π interaction with target molecules, resulting in energy approximately 20 kJ/mol [72]. Host-guest complexation is another type of non-covalent intermolecular bonding, which describes a selective interaction between host and guest molecules. Here, the host molecule contains a large cavity volume, which accommodates guest molecules. Guest molecule typically holds a complementary shape and reversible interaction with the host molecule, resulting in selectivity between the host and the guest [73]. When supramolecular materials fill in as host particles, the intensity of reversible interaction is significantly upgraded, hence appropriate for explicit acknowledgment of isomers. A delegate illustration of the host particle is cyclodextrin (CD) [74]. A regular CD ring comprises six to eight glucose units. Glucose units in all CDs are arranged such that the hydroxymethylene groups point downwards, while the hydroxyl groups point upwards, forming a hydrophilic outer space as well as hydrophobic inner space. Therefore, CD and their functionalized derivatives are suitable for improving the selectivity in an aromatic-involved pervaporation process.

Apart from considering different mass transport mechanisms individually, appropriate integration of these mechanisms is found to have the potential to maximize the separation performance of a rational-designed pervaporation membrane. There are mainly two ways of integrating mass transport mechanisms among which the first way is the physical integration of the molecular sieving mechanism and the solution-diffusion mechanism [41]. This helps in enhancing the solubility of molecular sieves and, moreover, improve the selectivity of hydrophilic materials. The second way is the physicochemical integration of facilitated transport mechanism with solution-diffusion mechanism [41]. Another way is the physicochemical integration of facilitated transport mechanisms with molecular sieving mechanism, which can be achieved in MMMs by embedding porous fillers with facilitated transport sites as well as size-exclusion nanochannels for permeate molecules [41]. All these result in an elevated performance of pervaporation separation.

## 3. Material Selection

The pervaporation membrane is one of the crucial factors in determining the overall efficiency of the separation process. A typical roadmap for membrane design generally starts from materials to preparation methods, structures, microenvironments, mass transport mechanisms and performance intensification [42]. However, there are several critical issues, which are to be addressed and considered when developing pervaporation membranes. This includes selectivity, productivity and stability. The chemical as well as physical properties of the pervaporation membrane and the interaction of the permeating species with the membrane should be emphasized during the realization of separation process. Therefore, the selection of an appropriate material to prepare pervaporation membranes is very relevant. 

**Figure 4:**
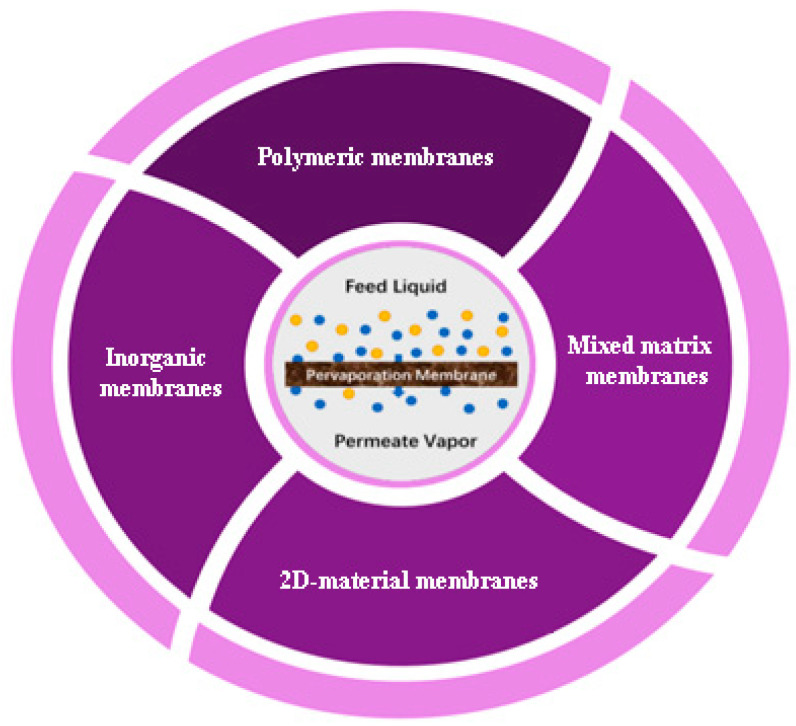
Classification of membranes.

Different classifications can be used for pervaporation membranes prepared from different starting materials and using various techniques, which are the physical blending method, hollow fiber spinning, in-situ polymerization, layer-by-layer (LbL) assembly method, sol-gel method, bioinspired methods, photo-crosslinking, solid solution casting and solution coating methods, described below in Section 4. In this review, the classification is based on the following types: polymeric, inorganic, 2D material and mixed matrix membranes (Figure 4).

### 3.1. Polymeric Membranes

Polymers are the largest family of membrane materials for pervaporation. Depending on the affinity, hydrophilic polymers are used to develop membranes for selective permeation of water over organics, and hydrophobic polymers—for organics. There is a large number of hydrophilic polymers for the preparation of pervaporation membranes such as polyvinyl alcohol (PVA), polyelectrolyte complexes (PEC), chitosan (CS), sodium alginate (SA), cellulose derivatives, polyamide (PA), polyimide (PI), and so forth.

Among this, PVA is one of the first commercialized pervaporation membrane materials [21], which remains as the benchmark polymer of hydrophilic membranes for solvent dehydration [43,75,76,77,78,79,80]. Moreover, blending PVA with other hydrophilic polymers with less compact structure has proved to reduce the crystallinity of PVA and thereby improving the membrane permeability. Dmitrenko et al. achieved the improved permeability of PVA membranes in the pervaporation dehydration of isopropanol by the introduction of 30 wt.% hydroxyethyl cellulose (HEC) or 20 wt.% chitosan (CS) into the membrane matrix [78,81]. The improved pervaporation performance of membranes for dehydration of isopropanol was achieved by blending of SA, one of the polysaccharides extracted from seaweed, with PVA [82]. It was shown an increase in permeation flux and a reduction in separation factor with the increase in the amount of PVA in the blend membranes. It means that the modified membranes generally suffer a trade-off between permeability and selectivity, and solutions to overcome this ended up in the fabrication of mixed matrix membranes by incorporating high performance fillers into the membrane matrix. In addition to dehydration, hydrophilic pervaporation membranes can be coupled with reactions. In the work [83] a catalyst was added to PVA casting solution to prepare a catalytic membrane to enhance reaction efficiency. The reaction occurred with the aid of a catalyst embedded in the membrane, and meanwhile the by-product water was removed by the PVA-based membrane via pervaporation.

Currently, biopolymers are the most alternative to chemically synthesized polymers in the manufacture of membrane materials. Among the biopolymers, CS, SA, cellulose derivatives are widely applied in the production of pervaporation membranes. Biopolymer CS derived from bacterial fermentation products is actively investigated for the separation of water-organic and organic compounds [84]. In pervaporation, the CS-based membranes are preferably employed and studied in hydrophilic pervaporation (the dehydration of organics) [85,86,87,88,89,90], since this polymer tends to permeate the more polar compounds, which is almost always water in a water-organic mixture.

SA obtained from vegetable sources is another potential biopolymer actively used for the preparation of pervaporation membranes for dehydration of organic solvents [91,92,93]. Especially important for environmental friendliness, when it is possible to create a blended membrane based on both biopolymers. In the work [94] novel membranes based on hydroxyethyl cellulose (HEC)/SA with improved transport characteristics were developed for pervaporation dehydration of isopropanol. The creation also of supported mixed matrix HEC/SA membrane by the introduction of fullerenol in a blend matrix allowed the achievement of the optimal transport characteristics in pervaporation dehydration of isopropanol (12–50 wt.% water): permeation flux of 0.42–1.72 kg/(m^2^h), and water content in the permeate of 77.8–99.99 wt.% [94]. The dehydration of other mixtures, which include ethanol, acetic acid, isopropanol, butanol, and so forth were also actively investigated in recent publications [95,96,97,98,99,100,101,102].

Cellulose acetate (CA) is another polymer for hydrophilic pervaporation membranes [103,104]. Series of blended PVA/CA membranes with different CA loadings were successfully prepared by Zhou et al. [105] and was employed to separate water-ethanol and methanol–MTBE mixtures by pervaporation. It was found that with increasing of CA loading, the hydrophilicity and the amorphous region of the blended membranes increased continually, along with the radius of the free volume cavity and the fractional free volume of the blended membranes. These blended membranes showed an improved pervaporation performance compared to the pristine CA and PVA membranes.

Polyelectrolyte complexes (PEC) are also a large family of hydrophilic polymers for the fabrication of pervaporation membranes for dehydration. PEC is formed when polyelectrolyte chains of opposite charge contact with each other at the interphase or in solution. Accordingly, there are two main types of PEC membranes: multilayer membranes fabricated by layer-by-layer (LBL) deposition of polyelectrolyte solution [106,107] and homogeneous membranes made by solution processable PEC [108,109]. The preparation method of solution casting of PEC membranes is more favorable compared with LBL approach for scalable fabrication. The first mentions of membranes from PEC on basis of weak polyelectrolytes were by Kalyuzhnaya R.I. et al. [110,111] in 1975. After there were works by Kikuchi Y. at al. [112] and Schwarz H.H. et al. [113]. 

PA membranes, prepared by interfacial polymerization between acyl chloride in organic phase and amine in aqueous phase, find its application in pervaporation, especially for dehydration of organic solvents [114,115,116,117], due to its thin film composite (TFC) polyamide layer and intrinsic hydrophilicity [118,119]. The most important for these membranes is the optimization of the conditions for their formation (concentration of amine and/or acyl chloride reagents, contact time, temperature and heating time, etc.) [116,120,121,122,123] in the process of interfacial polymerization. The TFC PA layer may also be used for the creation of membranes with a hierarchical structure. In the work [124], the supported CS/PAN membrane was improved by the formation of TFC PA layer by interfacial polymerization on its surface; this modified membrane had higher permeation flux in ~1.2–2.2 times maintaining high selectivity (99.9 wt.% water in the permeate) in pervaporation dehydration of isopropanol (12–100 wt.% water) compared to pristine membrane. In order to address the challenge of the dehydration of aggressive solvents at elevated temperature, PI with rigid chains such as polybenzoxazole [125,126] and polybenzimidazole [127,128,129] are used for the fabrication of membranes. These polymers possess thermally rearrange property that is useful for the tuning of molecular structure and thereby the membrane permeation properties. 

Hydrophobic polymers are another class of membrane materials generally used for selective removal of organic compounds from aqueous solution by the pervaporation. It is accepted in the scientific community that a polymer material is hydrophobic when its water contact angle is above 80° [130,131]. However, it should be noted that polyphenylene oxide (PPO) and polysulfone (PSf), which are hydrophobic since membranes based on them have a contact angle of water equal to or larger than 80 [132,133,134], are known to use dehydration due to transmitting small molecules in vacuum pervaporation (for example, water). The specific mechanism of mass transfer through these membranes can be described as follows: due to higher solubility and swelling organic substance interacts with polymers forming bonds and creating transport channels for water penetration [132,133]. PPO membranes have been already investigated for pervaporation dehydration of acetic acid [135,136], butanol [137], ethylene glycol [133,138], lactic acid [4], and so forth. The pervaporation dehydration of ethyl acetate [139], isopropanol [134], tert-butanol [140], ethanol [141], and so forth was carried out for the PSf membranes.

The hydrophobic membrane materials based on poly(dimethylsiloxane) (PDMS), poly[1-(trimethylsilyl)-1-propyne] (PTMSP), polyvinyl chloride (PVC), polymer of intrinsic microporosity (PIM-1), and so forth proved to be very effective in the pervaporation separation of organics from water [142]. PDMS is the most widely used hydrophobic membrane material exhibiting hydrophobicity, processability and stability in addition to excellent separation performance. There are two types of commercial PDMS precursors used for the development of pervaporation membranes. This includes hydroxyl-terminated PDMS that is often cross-linked by tetraethoxysilane (TEOS) and aminopropyl trimethoxysilane (APTMS) via condensation reaction to form a three-dimensional network structure [143,144]. The other is vinyl-terminated PDMS reacting with a hydrosilyl-containing cross-linker via addition reaction to form a linear architecture [43]. PDMS membranes were actively studied for the recovery of ethanol [145,146,147], butanol [148,149], isopropanol [142], furfural [150] from aqueous media, desalination of seawater and brines [151], and so forth, while PVC membranes were investigated for pervaporation recovery of propyl acetate, pentyl acetate, and octyl acetate from water [152], and PTMSP membranes—for alcohols [153,154,155,156]. Only from 2008 novel pervaporation PIM-1 membranes have been started to be studied for the recovery of methanol, ethanol and butanol [157,158,159,160,161,162,163,164,165], phenol [166] and ethyl acetate, dimethyl ether, and acetonitrile [33] from aqueous solutions.

Interest in the separation of organic–organic mixtures by pervaporation arose in the 1970s due to the need in the petroleum refining industry economically to separate benzene and aromatics from gasoline and diesel fuel, respectively [8]. Organic/organic mixtures are more challenging separation task for polymeric membranes than organic/water mixtures, because of the reduction in size discrimination ability and stability of the membrane due to much larger swelling of polymers in pure organic solution. Polymeric pervaporation membranes have been studied in separating three main kinds of organic mixtures: (i) aromatic/aliphatic such as benzene/cyclohexane and toluene/n-heptane; (ii) polar/non-polar such as methanol/methyl tert-butyl ether (MTBE); (iii) gasoline desulfurization such as model mixtures of thiophene/n-heptane.

Hydrophilic polymers in organic–organic separation are preferred for the recovery of polar components in polar/non-polar systems. Blended polyvinyl pyrrolidone (PVP)/polylactic acid (PLA) membrane were tested in pervaporation separation of ethanol-cyclohexane mixtures [167], PVA membranes were applied for the separation of methanol-toluene mixtures [168]. CA [169,170], PVA [171,172], polyamide-imides (PAI) [173] membranes and so forth were actively investigated for the pervaporation separation of methanol-MTBE, for separation of ethanol from ethyl tert-butyl ether (ETBE)—poly(pyrrolidinone) [174], and so forth.

Hydrophobic polymers, such as PDMS, PTMSP or polyoctylmethylsiloxane (POMS), are actively used for the selective separation of the less polar organics [8]. For example, PDMS membranes were investigated for the recovery of aromatics (toluene and benzene) from methanol [175]. There are a lot of another hydrophobic polymers used as a membrane material for pervaporation organic–organic separation such as PPO for separation of methanol—ethylene glycol [176,177,178], the methanol-cyclohexane [179], methanol-MTBE [180,181] mixtures; PVC for the separation of benzene-cyclohexane [182,183], toluene–n-heptane [184,185] mixtures; polyvinylidene fluoride (PVDF) for separation of methyl acetate-methanol [186] mixtures; and so forth.

Block copolymers with soft and hard segments such as polyether-block-amide (PEBA) [187] and polyurethane (PU) [188] are the most important high-performance membrane materials for separating organic/organic mixtures. The hard segment can inhibit excessive swelling, whereas the soft has an affinity for transporting organic substances. The size and chemical composition of the soft/hard segment can be adjusted to achieve comprehensive separation performance in terms of permeation flux, separation factor and structural stability in the organic/organic mixture. Among them, PEBA has attracted more attention because of its commercial production with variable soft-hard ratios and good processability [189]. PEBA has a higher affinity for aromatics such as benzene, toluene than aliphatics such as heptane or cyclohexane.

Thus, membranes based on low cost polymers with a high degree of scalability are still the dominant for pervaporation. However, in organic separation there is still a huge need for novel membranes, as existing modern polymers have problems such as lack of discrimination and low structural stability. In recent years, in the direction of dehydration the urgent development objective is to stabilize the structure of the polymeric membrane with a minimum decrease in performance and/or selectivity (to avoid the trade-off between permeability and selectivity).

### 3.2. Inorganic Membranes

Inorganic membranes with well-defined and rigid pore structures exhibit great separation performance and stability. They are generally prepared from crystalline microporous materials such as zeolite, covalent organic framework (COF), and metal-organic framework (MOF). Like polymers, inorganic membranes are also classified into hydrophobic and hydrophilic.

Zeolite being the first and the largest family of inorganic materials for pervaporation have tunable hydrophilicity and subnanometer size, which offer strong preferential adsorption, fast and selective diffusion to the intergrown crystalline membrane to realize efficient molecular separations [190]. Hydrophilic zeolites such as NaA, CHA, T-type are used for organic dehydration [191,192,193,194,195], whereas hydrophobic zeolites such as MFI can be used to fabricate pervaporation membranes for organic recovery (separation), whose water contact angle is about 103° [196,197]. Recently, many studies have focused on fabrication of thin and defect-free membranes. Firstly, Wang et al. proposed a dip-coating/wiping approach to deposit zeolite seeds in a uniform layer with highly reduced amount on the substrate, resulting in separation factor of 10,000 and flux of 9.0 kg/(m^2^h) for pervaporation dehydration of 90 wt.% ethanol/water mixtures at 75 °C [198]. This high flux was also attributed to the usage of ceramic hollow fiber substrate having low transport resistance.

MOFs, also known as porous coordination polymers (PCPs) or porous coordination networks (PCNs), are a new type of organic–inorganic hybrid membrane material based on coordination bonds between metal atoms or metal clusters (nodes) and organic ligands (linkers). They are also often utilized as fillers in MMMs [199]. Metal ions or metal clusters (so-called secondary build units—SBUs) are coordinated with organic ligands in MOFs, which are crystalline nanoporous materials. The strong bonds between a metal cluster and an organic ligand give MOFs a well-defined and stable structure. MOFs, as opposed to inorganic fillers, have a higher compatibility with the polymer matrix due to the presence of organic ligands in the structure [200]. The molecular sieving action and/or preferential adsorption mechanisms of MOFs to particular substances are used to achieve pervaporation separation utilizing MOF-based membranes. Certain molecules of a liquid mixture are prevented outside of the pores of MOFs, while others are allowed to pass through the pores of MOFs in the former mechanism. The pores of the MOF in the latter mechanism are large enough to allow all molecules in a liquid mixture to pass through [201]. Organic MOF ligands can also provide various interactions with separating substances [202]. In some cases, supramolecular interactions can occur between them, which can work when the geometry of the pores and the configuration of the penetrating molecules are combined to form certain channels or traps for a certain type of component, facilitating or hindering the separation. The crystalline membrane made of organic framework material with multifunctional pore structure and function is very suitable for pervaporation separation. Its prerequisite is to solve the water stability problem of MOF and reduce the inherent large pore diameter of COF. MOF is a new type of microporous materials, which require two prerequisites that should be met when applying pure MOF membranes for pervaporation separation: suitable pore size matching the permeant size and sufficient liquid stability [203]. Most MOFs are hydrophilic and suitable for organic dehydration, while a few of them are hydrophobic that can be used for organic recovery from aqueous solution [204]. The potential of pure membranes for the application in pervaporation separation of organic-inorganic mixtures was also explored for MOFs such as ZIF-71 [205,206], ZIF-8 [207], and MOF-5 [208]. The separation of organic–organic mixtures is primarily based on affinity and size sieving effect of MOFs. However, the separation performance of these MOF membranes is found undesirable compared with polymeric membranes. ZIF-71 membrane [205] showed dimethyl carbonate (DMC)/methanol separation factor of 5.34 that is comparable to that of PDMS membrane [209] but with 1–2 orders of magnitude lower permeation flux. Since the kinetic diameter of DMC (0.47–0.63 nm) is larger than methanol (0.36 nm), the selective permeation of DMC through ZIF-71 is mainly due to the preferential affinity towards less polar DMC molecules. ZIF-71 membrane synthesized via counter-diffusion method on a ceramic hollow fiber substrate had one order of magnitude higher permeation flux (2600 g/(m^2^ h)) with equivalent separation factor for 5 wt.% ethanol/water mixtures at 25 °C [206]. The abovementioned MOF membranes showed moderate separation performance as polymeric membranes, either for organic dehydration or organic separation, but much lower than zeolite membranes. Diestel L. et al. [207] investigated a supported ZIF-8 membrane in the pervaporation of n-hexane, benzene, and mesitylene liquids. It was shown that n-hexane and benzene permeated through the ZIF-8 membrane with the separation factor of 25, while mesitylene with larger molecular size (8.4 Å) could not permeate. Although, the bulky mesitylene is unable to enter the 0.34 nm sized ZIF-8 window despite with certain flexibility of MOF, the molecular sieving property for n-hexane/mesitylene could not be experimentally measured in ZIF-8 membrane due to the ultra-low permeation flux of mesitylene, which was close to the leakage of the apparatus. The permeation of n-hexane was hindered by the less mobile benzene in the ZIF-8 membrane but produced a moderate separation factor. MOF-5 membranes were tested in the pervaporation separation of pure o-xylene, toluene, and 1,3,5-triisopropylbenzene (TIPB) and their mixtures [208]. It was demonstrated that the permeation flux for the mixtures was lower than for the pure components. The maximum separation factors for toluene/TIPB and o-xylene/TIPB were 26.7 and 14.6, respectively. Fouling of MOF-5 membranes in the pervaporation separation of organic liquids was a critical issue. However, it also should be mentioned that water stability is an issue concerning most MOFs, which prevents their crystalline membranes from being used for the effective pervaporation dehydration of organic mixtures [210].

COF is another class of porous crystalline polymers. They are promising membrane materials due to well-defined and ordered pore channels [67]. COFs have higher stability in liquids compared to MOFs because of the covalent bonds between elements, such as H, C, N, O, B, and Si. There are three preparation methods of COF membranes: bottom-up strategy (interface-assisted synthesis and in-situ growth), and top-down strategy (blending and LBL technique) [211]. The pore sizes of COFs are in the range 0.5–4.7 nm depending on the geometry and the linkers [25]. Most COFs used as membrane materials are hydrophilic. Although, there are many available COF structures, only a few is converted into membranes. COF crystal pore sizes are not suitable for separating the small sized molecules involved in pervaporation separation, and to achieve an efficient separation, the various strategies are needed to reduce pore size of COFs. Firstly, Yang H. et al. [212] fabricated hybrid COF membranes by blending of COF and SNW-1 nanoparticles into sodium alginate (SA) matrix for ethanol dehydration. The obtained hybrid membrane with 25% COF had the high separation factor of 1293 and permeation flux of 2.4 kg/(m^2^h) in the pervaporation dehydration of ethanol (90 wt.%) at 76 °C. Since 2016, the active development and research of COF-based membranes has been started for the pervaporation dehydration of organic substances [213,214,215,216]. Membranes with COF were also tested in pervaporation of organic–organic systems, such as model gasoline desulfurization (thiophene/n-octane) [217] and toluene/n-heptane [218]. In the work [217] the porous COF nanosheets loaded with metal ions on their pore wall were blended with Pebax to prepare membranes with facilitated transport characteristics. The optimal membrane exhibited the separation factor of 6.29 and permeation flux of 21.6 kg/(m^2^h) for the separation of 1312 ppm thiophene/n-octane model gasoline at 60 °C. The thioether-functionalized hydrazone-linked COF (COF-LZU8) modified by Ag ion was incorporated into a commercial membrane based on poly(ether-block amide) (Pebax 2533) [218]. The Ag-COF-LZU8/Pebax membrane demonstrated a considerably improved performance: a permeation flux of 293 g/(m^2^h) and a separation factor of 4.03 in the pervaporation separation of 50 wt.% toluene/n-heptane mixture.

Thus, zeolite membranes are the most intensively studied, while the development of crystalline membranes from MOF and COF for pervaporation is still at an early stage.

### 3.3. Membranes Based on 2D Materials

Two-dimensional (2D) materials are as a new family of membrane materials since the discovery of graphene, which has received much attention [219]. It is expected a significant reduction in membrane thickness because of the atomic thickness of 2D materials causing fast component transport through the pores or interlayer channels of the membrane (increased permeability and selectivity of separation). Currently, pervaporation membranes from 2D material mainly used for dehydration of organic matter.

Among 2D materials for membrane separation, graphene-based materials has its own prior position as it has the advantages in single-carbon-atom thin and large lateral size up to hundreds of micrometers. Graphene can be fabricated either as a monolayer or a few-layered membranes or generally by chemical vapor deposition with/without the subsequent perforation process. Regarding the hydrophilic nature of graphene oxide (GO) nanosheets and the molecular sieving property of interlayer channels, GO membranes are well suitable for selective water separation from molecules with larger size and have been shown to be the most widely studied with excellent performance. The initial attempts on pervaporation GO membranes were conducted by Huang K. et al. [220] and Hung W.-S. et al. [221]. Supported GO membrane based on a ceramic hollow fiber prepared by a vacuum suction had the 95.2 wt.% water content in the permeate and a permeation flux of 1702 g/(m^2^h) in the pervaporation dehydration of 2.6 wt.% DMC/water mixture at ambient temperature (25 °C) [220]. In the work [221] GO membranes were prepared by the pressure-assisted self-assembly technique to deposit orderly flexible layers of GO on modified PAN substrate. This membrane demonstrated 99.5 wt.% water in the permeate and 2047 g/(m^2^h) permeation flux in the pervaporation separation of isopropanol (IPA)/water (70/30 *w*/*w*%) mixture at 30 °C.

Another type of promising membrane 2D materials is high-quality 2D COF, in which molecular transport can occur in both pores and interlayer channels [222,223]. The 2D COF membranes showed outstanding transport properties in pervaporation dehydration, which were even higher than for GO membranes: the separation factor of 3876 with permeation flux of 8.53 kg/(m^2^h) [222], separation factor of 4464 and permeation flux of 14.35 kg/(m^2^h) [223] for water/n-butanol (10/90 *w*/*w*%) separations at 80 °C. However, a very limited number of 2D COF membranes have been developed due to problems in synthesizing of defect-free large size nanosheets and their ordered stacking. In addition, the relationship between the structure and characteristics of pervaporation 2D COF membranes has not yet been studied.

In addition, MXene and layered double hydroxide (LDH) are the other 2D materials for the development of pervaporation membranes for organic dehydration. MXene is a new family of 2D transition metal carbides and carbonitrides, applied as building blocks of membranes for gas separation [224,225], pervaporation [226,227,228], desalination [229], and water purification [230]. It has large variety of structures due to different transition metals (more than 30 synthesized compositions) with improved mechanical stability compared to graphene-family materials. In most investigations, MXene nanosheets have been introduced into pervaporation membranes. The first works on pervaporation membranes with MXene were presented by Liu G. et al. [227] and Xu Z. et al. [90]. Ultrathin MXene membrane onto the commercial PAN ultrafiltration substrate developed by stacking synthesized atomic-thin MXene nanosheets was tested in pervaporation desalination at 65 °C: water flux of 85.4 L/(m^2^h) and salt rejection of 99.5% with feed concentration of 3.5 wt.% NaCl [227]. Newly developed MXene/CS membrane used for solvent (ethanol, ethyl acetate and DMC) dehydration via pervaporation at 50 °C demonstrated improved performance: ~1.4–1.5 kg/(m^2^h) permeation flux and 1421, 4898 and 906 separation factor, respectively [90]. So far, the performance of MXene membranes requires much more investigations to the control of interlayer space (the nanochannel size) and eliminate defects in membranes [231]. It should also be noted that MXene is subjected to degradation under humid conditions—in the presence of oxygen, MXene metal atoms on the surface are prone to spontaneous oxidation. To overcome these limitations various modifications of Mxene have been developed. Despite the great potential of LDH consisting of positively charged brucite-like laminates with charge compensating anions [232], it remains challenging to fine-tune the orientation and the interlayer galleries size in the membrane [25].

### 3.4. Mixed Matrix Membranes

In addition to the pervaporation membranes from a single material type, mixed matrix membranes (MMMs) are prepared by introduction of various fillers into a polymer matrices and have attracted a tremendous attention since the 1990 [63]. This approach allows solving the trade-off problem of permeability-selectivity by proper selection of optimal modifiers [64], which may significantly change structural and physicochemical characteristics (surface functionalization, change of free volume, morphology, etc.) causing improved transport properties [94,98,233,234,235,236]. Depending on the separation task, hydrophobic or hydrophilic fillers may be used to prepare MMMs enhancing the membrane adsorption towards water or organics [237]. The development of MMMs is in step with the development of nanomaterials that are actively used as fillers at present. In 1990–2010 the first MMMs were created based on inorganic fillers such as zeolites and silica. However, they could not exhibit a high performance due to the challenges in filler dispersion and interfacial voids [238]. Compared with inorganic fillers, since the 2010 the advancement with nanomaterial fillers such as MOFs [100,102,239,240,241], 2D materials [242], and so forth with tunable organic groups show much better compatibility with polymers due to diverse functionalities and structures, resulting in highly enhanced dispersion and interfacial morphology in membranes [243]. The first works on the creation of pervaporation MMMs by the introduction of hydrophobic MFI zeolites into PDMS membrane were conducted by Sano T. et al. [244] and H.J.C. te Hennepe et al. [245]. However, to date, the combination of polymers and zeolites in MMMs has not been as successful for pervaporation because of the trade-off problems of a uniform filler dispersion—high filler loading, an interfacial defect—chain stiffness. In the future, great attention is needed to pay to new synthesis approaches of zeolite nanoparticles and the create of favorable interactions it with the polymer matrix [25].

The second generation of fillers such as MOFs, COFs and 2D-materials, and so forth could solve these problems: nanoparticle synthesis, their uniform dispersion, interfacial voids and thick membrane layer without defects. Choosing a suitable MOF as a modifier, it is necessary to take into account its effect on sorption, affinity, and diffusion, pore size, hydrophilicity, and its liquid stability. The most studied MOF for pervaporation membranes is ZIF-8 easily synthesized at ambient temperature with high yield [128,239,246,247,248,249]. The hydrophilic MOFs (UiO-66 and MOF-801) are more suitable modifiers for hydrophilic MMMs [100,250,251,252,253]. MMMs with MOFs have shown clear advantages over MMMs with zeolites in the synthesis of nanofillers, their uniform distribution in the polymeric matrix, and the possibility of creating a thinner selective membrane layer. However, to date, only a small amount of MOFs has been used as a modifier for pervaporation membranes.

COFs with versatile pore structure and crystalline nature showed remarkable performance enhancement for polymers and have higher stability with lower molecular sieve capacity compared to MOFs [67]. The introduction of COFs into membrane may significantly improve the pervaporation characteristics due to their preferential adsorption capacity and diffusion channels. Hydrophilic COFs (TpHz and SNW-1 [212,254]) are used as fillers to improve the water permeability and selectivity of polymeric (poly(ether sulfones (PES) and SA, respectively) membranes, while the introduction of COFs such as hydrazone-linked COF-42, COF-300 into PDMS [215,255] and hydrazone-linked COF-LZU8 modified with Ag into Pebax 2533 [218] membranes led to selective permeation of organics from mixtures. COF fillers have shown great potential in both hydrophilic and hydrophobic membranes, causing the enhanced pervaporation performance. However, the effect of COFs as fillers on the mass-transport mechanism is needed to be understanding in the detail.

Graphene-based materials are the first and mostly accepted 2D fillers for polymeric MMMs. GO is the most studied 2D material due to easily synthesized single-layer nanosheets and its oxygen-containing groups. Its structure allows the achievement of good dispersion and compatibility with the polymer matrices, surface functionalization, and preferential sorption centers. GO can also be functionalized with hydrophobic groups and has been used as fillers for hydrophobic MMMs. Thus, this modifier can be used both for dehydration [133,256,257,258,259] and for the extraction of organic substances from an aqueous solution [164], and the separation of organics [260]. The introduction of GO significantly increase the efficiency of dehydration in polymeric MMMs. However, although it was reported that GO is an amphiphilic material with hydrophilic edges and a hydrophobic base, the original GO does not have good organic permeability. Therefore, in many studies, modification or functionalization of GO are carried out. For example, modification with ionic liquid to incorporate into PEBA membranes for the pervaporation of butanol aqueous solutions [261], with octadecylamine (rGOODA) to incorporate into PDMS membranes for the removal of toluene from water [262], with spirobisindane to incorporate into polyimide (Matrimid 5218) membranes [263], with Ag nanoparticle to incorporate into PI membranes for separation of benzene/cyclohexane mixture [264], and so forth were carried out. However, the transport channels within these MMMs are still not clear because of the presence of the non-porous or porous filler structure.

The advancement of membrane materials plays a crucial role in progressing the development of pervaporation. Polymeric membranes are still the dominant membrane materials for pervaporation due to their advantages: low-cost, high scalability, easy fabrication, structure stability, and so forth. The inorganic membranes inferior to polymeric membranes due to high price, relatively rigid structure, low acid stability, and the difficulty of processing [24]. The properties of membranes from 2D materials are more similar to inorganic membranes with well-defined pores. MMMs with uniform modifier dispersion and ideal interface have balanced pervaporation performance compared to pristine polymeric and inorganic membranes. However, it is necessary to establish predictive models for MMMs and gain more theoretical understanding on realistic mass transport within new 2D and 3D materials. Organic materials with multifunctional chemical groups, inorganic materials with well-defined hierarchical structures and hybrid materials combining the advantages of both materials should be actively developed. Nowadays, most of the research is aimed at studying ways to stabilize the membrane structure and properties with minimum productivity decline, to reduce the thickness of separation layer, to understand the effect of substrate material and interface on the membrane performance, and to combine advantages of various membrane material types.

## 4. Fabrication Techniques

**Figure 5:**
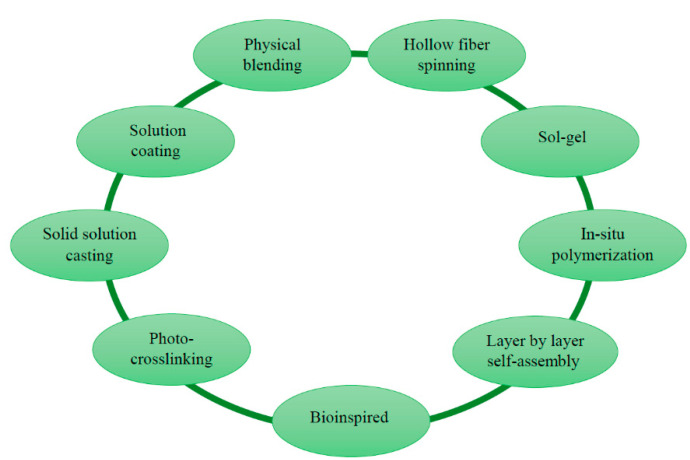
Commonly used fabrication techniques.

For the fabrication of pervaporation membranes, there are many methods that can be divided into physical and chemical [265]. Among the common methods for developing membranes, the following can be distinguished: the physical blending method, hollow fiber spinning, in-situ polymerization, layer-by-layer (LbL) assembly method, sol-gel method, bioinspired methods, photo-crosslinking, solid solution casting and solution coating methods. Figure 5 is a schematic representation of methods for fabrication of pervaporation membranes, which are covered in this review.

**Physical blending method** is widely used among the physical methods. Various fillers can be used in the physical blending method, such as silicon dioxide nanoparticles [266], GO [267], metal oxide nanoparticles [268], CNTs [269], MOFs [270], and so forth. In physical blending, fillers are physically dispersed in the polymer matrix by mixing a solution, an emulsion, or a melt until homogeneous mixtures. The mixture of polymer and filler is then cast onto a porous support, and hybrid membranes are obtained after complete evaporation of the solvent. Cha-Umpong et al. [267] developed a polypropylene (PP) membrane with GO by filtration through a PP-based hollow fiber membrane under vacuum different volume of 0.02 mg/mL GO suspension to obtain a GO layer of various thicknesses. Metal oxide nanoparticles are potential fillers in fabrication techniques as these nanoparticles with rich functional groups and high specific surface area make it possible to spread within the polymer matrix homogenously, and hence prevent any voids or pores at nanoparticles or polymer matrix interface. Pervaporation membranes were made using a chitosan membrane containing iron oxide nanoparticles by Dudek et al. [271]. CNTs are another type of material, which can practically be used in physical blending methods. However, pristine CNTs are chemically inert and hence cannot mix homogenously into the polymer matrices. This may cause agglomeration, which can be prevented by using modified forms of CNTs. Gao et al. [272] carried out a study using multiwalled carbon nanotubes (MWCNT), in which Fe_3_O_4_ nanoparticles were attached to MWCNTs and then strongly incorporated into SA to obtain SA-Fe_3_O_4_@CNT hybrid membranes. This improved their dispersion in the membranes and, as a result, the overall performance.

**Figure 6:**
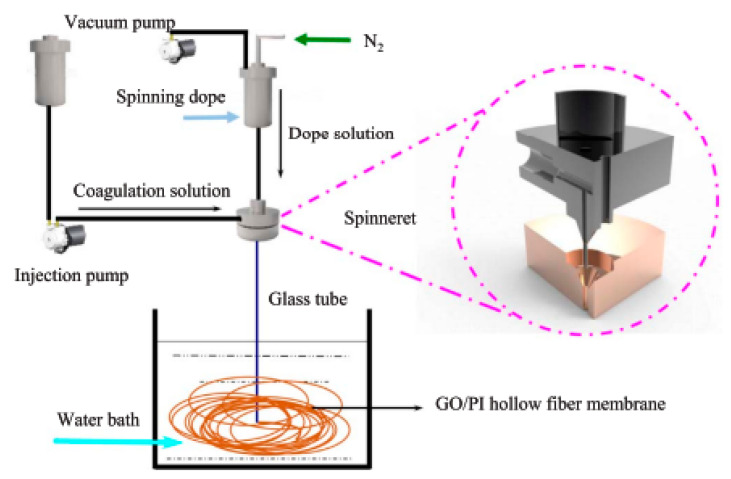
Schematic of the preparation of GO-PI hollow fiber membranes by direct spinning. Reprinted with permission from Ref. [274]. Copyright 2018 Aisheng Huang et al.

**The hollow fiber spinning method.** Relative to a flat membrane, a hollow fiber membrane has the advantages of self-supporting structure, a self-contained vacuum channel and high filling density. In the spinning process, the membrane is formed by phase transformation, when the primary fiber contacts with coagulant. Extruding the polymer coating and the liquid in the inner hole of the primary fiber at the same time, the primary fiber immediately solidifies on its inner surface. Apparently, due to the air humidity, as the primary fiber passes through the air gap region, a part of it solidifies on the outer surface. The complexity of hollow fiber spinning is increasing with the development from single-layer to double-layer co-extrusion. This is the cost-effective membrane preparation method and more optional in the choice of materials and forms of the support layer. For instance, Tsai et al. [273] scrutinized a novel fabrication method of hollow fiber PA/PAN membranes, using a triple orifice spinneret. Trimesoylchloride (TMC) and tetraethylenepentamine (TEPA) were used as the monomers of acid chloride solution and aqueous solution, respectively. The PAN dope, TEPA and TMC solutions were injected into the outermost, middle, and inner channel of the triple orifice spinneret, and simultaneously co-extruded into the water. Then, a PA layer was formed on the lumen surface of the synchronous wet-spun PAN membrane. Huang et al. prepared an asymmetric GO-PI hollow fiber membrane by direct spinning of a GO/PI suspension via phase inversion in water/NMP coagulation (Figure 6) [274]. In the process, GO aqueous suspensions were prepared by the modified Hummers’ method using graphite powder.

**The sol-gel method** is one of the common chemical methods, which is widely used to prepare hybrid membranes due to its eco-friendly nature and low temperature requirement. Here, an inorganic precursor, such as silica, is dispersed in polymer solution at a favorable temperature. Sol-gel reactions involve hydrolysis of the inorganic precursors as well as subsequent solidification of polymer chains. The kinetics of the sol-gel process is a significant factor in processing an ideal hybrid membrane. This is because the hydrolysis and condensation rate of the precursor and solidification rate of the polymer chains should be compatible. To obtain an ideal sol-gel processed membrane, several factors such as temperature, precursor types, catalysts, and so forth need to be manipulated [265]. Zhou et al. [275] prepared a membrane by dissolving PDMS (crosslinker:prepolymer 10:1 wt.%) and silicate-1 in n-heptane. Then the platinum-cure was added to the polymer solution. The membrane was formed by depositing a polymer layer on a PVDF substrate. The versatile functionalities of silica substances have been the center of attraction of many researchers. H. Zhou et al. [276] worked on superhydrophobic organo-inorganic hybrid membranes. PDMS was dissolved in heptane followed by the addition of 10–20 wt.% silica. After mixing, the membrane was formed using a casting machine. A good compatibility was observed between the inorganic and polymeric components. This attributed to the formation of covalent bonds between the two phases.

**Figure 7:**
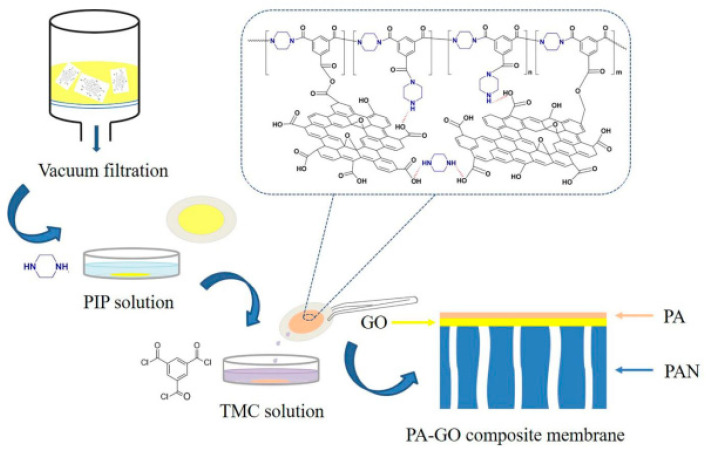
Preparation of PA-GO composite membranes via pressure-assisted ultrafiltration with subsequent interfacial polymerization. Reprinted with permission from Ref. [279]. Copyright 2020 Liu X. et al.

**The in-situ polymerization method** is another common chemical method. If the polymer chains and the inorganic precursors are not compatible, it can pose challenges in fabricating hybrid homogenously dispersed membrane. In-situ polymerization method can tackle the issue. In this method, the polymer monomers along with the inorganic soluble precursors are dissolved in a certain solution, such as alcohol and others. Under favorable conditions, the precursors can initiate aggregation of polymer monomers through physical or chemical interactions. After surface modifications, the inorganic particles can be dispersed homogenously in the matrix. Due to their relatively lower molecular mass, they can add a steric effect to the polymer, which enhances its stability and thereby the quality of the membranes. The main disadvantage of this method is that the intercalation of polymer chain is restricted. Therefore, proper dispersion of the filler requires appropriate modification of the particle surface [277]. Li Y. et al. [278] prepared pyromellitic dianhydride (PMDA)-4,4′-oxydiphenylene diamine (ODA) PI membranes with non-macrovoid structures. The following procedure was carried out: equal moles of PMDA and ODA were reacted in N-methyl pyrrolidone (NMP) in an ice bath for around 6 h. Then, tetrahydrofuran (THF) and glycerol (GLY) were added into the solution. The membrane was prepared by the polymer solution casting on a glass board followed by immersion in ethanol/water bath at ambient temperature during 30 s. This would inhibit the formation of macrovoids. The membrane then was dried using n-hexan-isopropanol solvent displacement method to avoid hydrolysis. Later, the membrane was subjected to imidization via thermal or chemical processes. The use of PA membranes for pervaporation desalination has also been explored by Zhao X. et al. as a replacement to reverse osmosis for seawater desalination [279]. The modifier was prepared according to the following procedure: GO powder was dispersed in distilled water, and then centrifugation separating of non-stratified and stratified sheets of GO (supernatant) was carried out. The supernatant (0.1 mg/mL) was used to prepare composite membranes. The supernatant was filtered through the PAN membrane, and then in-situ polymerization was carried out between piperazine (PIP) and benzenetricarbonyl trichloride (TMC). Preparation of PA-GO composite membranes is shown in Figure 7.

**Layer by layer self-assembly** is a common method for both membrane preparation and surface modification. This method often shows a “sandwich-like structure”. It is a cyclic process, where a charged material is adsorbed onto a substrate, after rinsing with a oppositely charged material. It gets adsorbed to the top of the first layer. The dispersion process can be repeated until the desired thickness will be obtained. Interactions between the layers include covalent, hydrogen bonds, electrostatic interactions, and so forth. In most cases, the membranes formed via this method can attain high flux, which is due to the ultrathin separation layers. Zhang et al. [280] reported new hybrid membranes using an amphoteric oxide nanoparticle-controlled ex-situ layer by layer self-assembly. The oppositely charged particles could be produced by adjusting the charge property of amphoteric oxide nanoparticles in acidic and basic conditions. They used poly(sodium styrene sulfonate) (PSS) and poly(diallyldimethylammonium chloride) (PDDA) as polyelectrolytes and ZrO_2_ particles as nanoparticles. The charge property of nanoparticles had a significant role in deciding the stability of the suspension. The PSS-coated ZrO_2_ and PDDA-coated ZrO_2_ nanoparticles were then used as building blocks of nanohybrid multilayers. In a study of Chaudhari et al. [281] the membranes were prepared by covalent PVA-TEOS cross-linking using a GO/CS polyelectrolyte layer to enhance the surface of membrane by LBL interfacial complexation. LBL approach using CS and GO improved the separation efficiency and flux.

The works of Dmitrenko et al. [78,79,282] demonstrated that the simultaneous use of bulk and surface modifications is a promising way to create highly efficient membranes for pervaporation dehydration of isopropanol. Thus, PVA membranes were developed using bulk (introduction of chitosan, fullerenol, and poly(allylamine hydrochloride)) and surface modifications (by LBL assembly using various polyelectrolyte pairs of poly(sodium 4-styrenesulfonate)/poly(allylamine hydrochloride), poly(sodium 4-styrenesulfonate)/chitosan, polyacrylic acid/chitosan). It was shown that a correctly selected polyanion-polycation pair, the number of cycles, the order of their deposition by LBL, as well as additional bulk modification (the introduction of poly(allylamine hydrochloride), chitosan, and fullerenol) have a significant effect on the transport characteristics of membranes. The best developed membranes for separation of water/isopropanol (20/80 wt.%) mixture were: (i) a chemically cross-linked supported PVA membrane with 4.7 wt.% poly(allylamine hydrochloride) and 5 wt.% fullerenol in the bulk and 10 bilayers of poly(sodium 4-styrenesulfonate)/poly(allylamine hydrochloride) on the surface; (ii) chemically cross-linked supported PVA membrane with 5 wt.% fullerenol and 20 wt.% chitosan in bulk and 5 bilayers of poly(sodium 4 -styrenesulfonate)/chitosan on the surface. The chemical cross-linking was carried out by adding 35 wt.% MA and heating at 110 °C for 120 min. The developed membranes had a permeation flux of more than 8.4 times that of the commercial membrane “PERVAP^TM^ 1201”. It was shown that polyelectrolyte layers on the surface of supported membranes with a nonporous selective PVA layer were not washed off during pervaporation dehydration and when the membrane was kept in water for a long time.

Halakoo E. et al. [283] experimented on LBL self-assembled membranes from polyetyleneimine (PEI) and GO. It was found that LBL deposition of PEI/GO on a chlorinated TFC PA substrate under electrostatic interactions and hydrogen bonds between ionized carboxyl groups in GO and protonated groups in PEI displayed remarkably good permeation flux, selectivity and overall performance. On the other hand, presence of salt in the feed solution lessened the permeation flux. However, flux can be controlled by various methods such as a change in the membrane module design to surface area enlargement, reducing the active layer thickness, change in the experimental parameters like temperature, and so forth. Thus, the modification of LBL membranes to enhance the flux and overall performance of the membrane would result in the fabrication of future membranes with massive potential.

**The bioinspired method**. The method of induction of inorganic precursors to be mineralized by synthetic or biological molecules in vitro has received broad attention. This method can generate inorganic nanoparticles as nanofillers in the polymer matrices of hybrid membranes at mild conditions. The precursors used are often alkoxide molecules or inorganic salts, and the inducers used are synthetic or biological molecules. Their interactions regulate the formation of inorganic particles at the molecular level. Li B. et al. [284] processed PDMS/SiO_2_ hybrid membranes through the controlled biomimetic mineralization in confined space, using NH_3_ or cysteamine as inducers and TEOS or tetramethylsilane (TMOS) as silicon precursors. TEOS or TMOS, being highly reactive, also acted as the cross-linking agents for PDMS, which were favorable for creating the free volume property of the hybrid membranes. Moreover, the compatibility between the PDMS matrix and the SiO_2_ inorganic particles enabled the homogeneous dispersion after mineralization. In biomimetic mineralization, the inorganic precursors are mixed with polymers at the molecular level and the space of polymer network is limited. This contributes to the in-situ formation and homogenous distribution of nanoparticles. Meanwhile, the process is conducted under mild conditions, (ambient temperature and pressure, pH ≈ 7.0) the membrane materials can degrade by strong acid, alkali, or high temperature.

**Figure 8:**
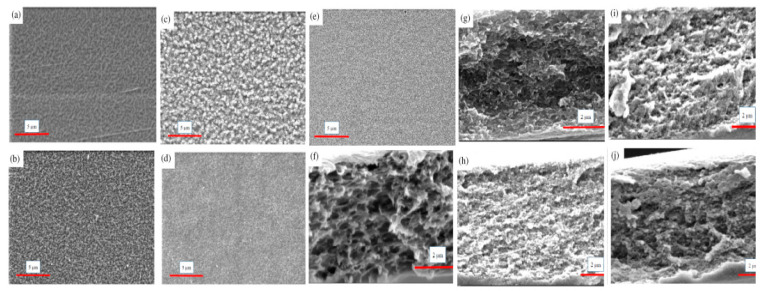
SEM surface images of (**a**) CTA pristine, (**b**) CTA-1 wt.% SiO_2_, (**c**) CTA-2 wt.% SiO_2_, (**d**) CTA-3 wt.% SiO_2_, (**e**) CTA4 wt.% SiO_2_ membranes, SEM cross-sectional images of (**f**) CTA pristine, (**g**) CTA-1 wt.% SiO_2_, (**h**) CTA-2 wt.% SiO_2_, (**i**) CTA-3 wt.% SiO_2_, and (**j**) CTA-4 wt.% SiO_2_ of membranes. Reprinted with permission from Ref. [286]. Copyright 2020 Prihatiningtyas I. et al.

**The solid solution casting method** is the most commonly used method for the synthesis of flat membranes applied in various situations [237,285]. The polymer and potential additives such as inorganic fillers are dissolved in a solvent first to form a solution and then the mixtures are shed onto a flat surface such as a stainless steel plate or a Petri dish. The solvent is usually removed through evaporation or phase inversion. Through the casting method, the multilayer film can be successfully prepared. A high-density cell membrane can be prepared by removing the solvent slowly and fully through evaporation. In contrast, an asymmetric membrane can be obtained by immersion in a non-solvent bath, where the solvent removal process includes a phase inversion process. An effective way to achieve the purpose of preparing the top dense layer is the addition of a highly volatile solvent to the casting solution and performing phase inversion after evaporation. When preparing MMMs, the mixtures are commonly treated by sonication and thorough stirring to prevent the agglomeration of fillers. Prihatiningtyas et al. prepared nanocomposite pervaporation membranes produced through solution casting from materials such as cellulose triacetate (CTA) and commercially available colloidal Lu-dox-SiO_2_ nanoparticles [286]. CTA was chosen as polymer matrix due to its high salt rejection, excellent mechanical and chemical properties, low cost and fouling tendency, and the fact that it could be processed to yield dense films. Meanwhile, silica nanoparticles were selected as nanofillers due to their attractive properties such as low toxicity, hydrophilicity, and excellent mechanical properties. Membranes with different combinations of CTA/LUDOX-SiO_2_ were developed and characterized to evaluate the pervaporation efficiency. A number of LU-DOX AS-40 suspensions were prepared, varying SiO_2_ wt.% (1, 2, 3 and 4 wt.%) in dope solutions. LUDOX AS-40 is a commercial suspension with 40 wt.% silica in water. The LUDOX dispersion was stirred at 70 °C for 3 h at 1000 rpm. 6 wt.% of CTA was added to it and stirred for another 3 h at 70 °C. The solution was sonicated for the next 2 h, stirred overnight and was casted onto glass plates at room temperature. So, dried membranes were immersed in a water bath. Figure 8 shows scanning electron microscopy (SEM) micrographs of the membranes developed in [286].

SEM images (Figure 8) of the membranes show that pristine CTA adopts a sponge-like structure with dense top and bottom layers. An addition of 3–4 wt.% of SiO_2_ caused a change in the cross-section membrane morphology. The study reports that SiO_2_ NPs filled the pores in the sponge-like structure of CTA and resulted in a uniform dense structure. On evaluating the performance of the membranes with a feed solution with a concentration of 30g/L NaCl, it was investigated whether increasing the concentration of SiO_2_ NPs in the CTA caused an increased water flux without compromising the salt rejection. Salt rejection stayed above 99%. Best performance was obtained for the membrane with 4 wt.% SiO_2_, due to the increased hydrophilicity caused by the SiO_2_ NPs. A further addition of SiO_2_ NPs would lead to a decrease of the mechanical strength of the membrane. The high performance of CTA/4 wt.% SiO_2_ was also due to non-volatility and low diffusivity of the NaCl solution.

**The solution coating method** is commonly used to deposit a thin selective layer on a microporous substrate to prepare a supported membrane. These substrates can be flat, hollow fiber or tubular form, but they must be completely porous to have low resistance. The pore size distribution of the substrate surface should be dense without large defects, which should prevent the coating solution invasion. Before coating, the substrate is pre-wetted by a solvent with a low boiling point, and which is not compatible with the coating solvent. This minimizes the risk of intrusion. Hence, to obtain the coating film, the pre-wetting solvent is removed by drying. It is a big challenge evenly to coat the hollow fiber, as uneven coating on the small-diameter hollow fiber negative effects on the separation [287].

The use of pervaporation membranes in aqueous solutions often requires **cross-linking of the polymer chains**. Currently, many different cross-linkers and methods are used, in some cases cross-linking occurs when polymer films are heated [76], sometimes a cross-linker is added to the polymer solution with the following heating [77,78]. There are cross-linkers that do not require additional processing at elevated temperatures, for an example, glutaraldehyde [239]. Sometimes it is enough to immerse the developed membrane into a solution with a cross-linking agent [98,102]. In recent years, one of the developing areas of cross-linking of polymer chains is photo-crosslinking. **Photo-crosslinking** of polymer chains is an efficient and sustainable approach without the use of hazardous/toxic reagents. In this method, the cross-linking of polymer chains occurs due to UV influence both on the polymer itself and on its additives. For example, a TFC membrane that comprises a photo-crosslinking PVA-stilbazol quaternized (SBQ) dense selective layer for desalinating brine solutions containing up to 20 wt.% NaCl was developed by J. Meng et al. [288]. PVA-SBQ is first dissolved in deionized water to form a 15 wt.% dope solution. The PVA-SBQ films were fabricated by knife-casting of the dope solution on a glass support. The dope layer thickness was 25 μm and 200 μm. Dry polymeric membranes were cross-linked using UV irradiation (365 nm) for various time (5 s, 30 s, 1 min and 10 min) at different light intensities. Under the influence of UV, SBQ can dimerize on the membrane surface with the formation of a quaternary ring, which leads to the cross-linking of PVA chains [289].

Thus, depending on the polymer, modifier and separation task, different approaches for fabrication techniques of pervaporation membranes can be chosen.

## 5. Characterization Techniques

Characterization is an important area of membrane technology that focuses on studying the properties of a membrane material for better understanding of mass transfer through membranes. The characterization allows the optimization of the characteristics of membrane materials and is widely used to confirm the quality and purity of prepared membranes and to interpret membrane characteristics [290]. It is also worth noting that characterization methods of MMMs provide valuable information about the interaction between matrix and fillers. The characterization of materials and membranes based on them includes various analysis methods to study: (1) physical and physicochemical properties by investigation of tensile strength, film thickness and density, moisture content, swelling properties, chemical resistance, thermal stability, contact angles, and so forth, (2) structural properties that make it possible to analyze the structure of membranes at various scales by X-ray structural methods, spectroscopy, optical, microscopy methods, and (3) transport properties by investigation of sorption and diffusion selectivity, permeation flux and permeability, separation factor, permeance, concentration factors, and so forth.

The physical and physicochemical properties of membranes include various aspects and directions, and are investigated by various methods. The mechanical properties for membranes and films are usually determined by the investigation of the tensile strength and Young’s modulus [99,100,291,292]. Membranes in the practical use are exposed to various external forces, because of which it is also necessary to study their mechanical strength [291]. If they do not meet the basic requirements of its application, then the membranes will be largely limited. Due to the stages of pervaporation mechanism solution-diffusion (component sorption, diffusion through the membrane (in particular, due to the free volume between the polymer chains) and desorption from the other membrane side), it is also important to investigate the properties of surface, affinity to the feed components and density of the membranes. In this regard, parameters such as membrane swelling degree (sorption), contact angles and density are investigated, which allows estimating the component transport through the membranes [81]. The density of the pervaporation dense membranes are usually determined by flotation method [81]. The investigation of swelling degree (sorption) provided by gravimetric method allows to evaluate membrane stability in components and mixtures, to predict the components separation, as well as the amount of desorbing and physically sorbed solvent by the membranes [235,240]. To evaluate the hydrophilic–hydrophobic balance of the membrane surface, the contact angle values usually obtained by the sessile drop method are used [133,293], since changes in surface properties largely affect the first stage of the pervaporation mechanism. All these obtained parameters allow an explanation of the mass transfer of low molecular weight substances in pervaporation through the membranes. In addition, the thermal stability of membranes is also a key factor in the recipe for the success of high temperature membranes. The concept of thermostable membranes includes three critical factors that determine their ultimate utility: stability, durability and manufacturability [294]. As a rule, membranes in industry are subjected to prolonged operation at elevated temperatures to accelerate separation processes. The membranes must exhibit high thermochemical properties and a favorable ratio between selectivity and permeability [294]. Thus, the thermochemical properties of membrane materials are usually investigated by thermogravimetric analysis (TGA), which makes it possible to evaluate not only the temperature limit of the membrane application, but also the effect of modifications on its structure and stability.

The most common investigation techniques of structural properties are used to study morphology, crystal structure, functional groups, and chemical composition of membrane materials [290]. Fourier-transform infrared (FTIR), Raman and nuclear magnetic resonance (NMR) spectroscopies are generally used to determine the chemical composition, formation of novel interactions and functional groups. To study the inner and surface morphology of membranes, scanning electron microscopy (SEM), atomic force microscopy (AFM) and transmission electron microscopy (TEM) are applied. X-ray diffraction (XRD) is classically used to investigate the crystal structure, its shape and size, while small-angle and wide-angle X-ray scattering (SAXS and WAXS) methods provide crystallographic data with additional information about particle size and pore size distribution. In the review [290] each of these methods are discussed in terms of application, preparation, advantages and limitations for membranes.

Transport properties of membranes, of course, are evaluated in pervaporation separation of various mixtures, the choice of which depends on the separation task and the selected membrane material. Transport parameters are usually calculated based on the obtained data during pervaporation experiment (weight of permeation, permeate composition, time of permeate collecting, etc.) and presented in terms of permeation flux and separation factor [295]. However, Baker R.W. illustrated the benefits of driving force normalized properties [66]. His paper reported how to present performance data of pervaporation membranes as intrinsic, driving force normalized properties in terms of permeability, permeance and selectivity rather than as permeation flux and separation factor. The problem with pervaporation data in terms of permeation flux and separation factor is that these values are not only a function of the intrinsic membrane properties, but also significantly depend on the operating experiment conditions (permeate pressure, feed concentration and temperature, membrane thickness, etc.). Thus, the use of these parameters makes it difficult to compare pervaporation datasets obtained under different conditions, while permeability, permeance and selectivity allow universally compare the membrane performance [66]. The sorption and diffusion selectivity can be expressed from the membrane separation, which are intrinsic membrane material properties, although, sorption selectivity may depend on the characteristics of the solution [296].

Thus, the characterization of membranes are a powerful tool for studying the structure, physicochemical properties, and transport characteristics. The data obtained are used to design and optimize membranes to improve their performance.

## 6. Applications

### 6.1. Organic–Organic Separation

**Figure 9:**
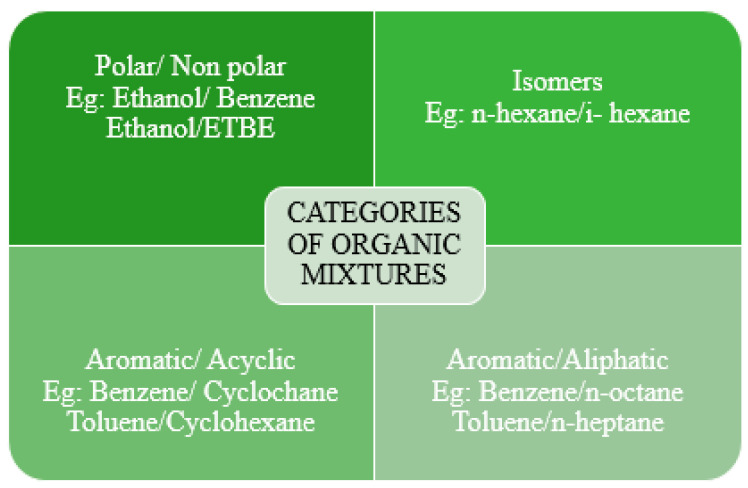
Classification of organic–organic mixtures.

Distillation is the most common method for the separation of organic mixtures in the petroleum and chemical processing sectors. Distillation is an energy-intensive process that accounts for around 40% of the total energy utilized by the chemical processing in-dustries. Refiners seeking to recover aromatics such as toluene and styrene from various heavy, intermediate, and light catalytic naphtha streams created the first organic–organic issue. They also intended to lower the amount of benzene in the C6 reformates used to make high-grade lead-free gasoline. The synthesis of MTBE and ETBE, which are commonly used anti-knock substitutes for tetra-ethyl-lead to increase the octane number of gasoline, is primarily connected to the fractionation of alcohols/alkanes and alcohol/ether mixtures. As a result of their azeotropic characteristics, alcohol–ether mixtures are difficult to separate. Removal of aromatics from the feedstock of ethylene plants to increase production capacity and removal of cyclohexane from benzene/cyclohexane mixtures created in benzene, toluene, and xylene manufacturing plants are two possible applications of aromatics/saturates separation in the chemical industry [107]. Separation of organic–organic compounds is one of main areas in which pervaporation can be most useful. Different organic compounds either have a close-boiling point or form azeotropic mixtures, which cannot be efficiently separated by techniques such as distillation [108]. Separation of close-boiling organic–organic mixtures using distillation or liquid–liquid extraction is tough as the compounds generally have similar physical and chemical properties with respect to one another. Pervaporation can be used to separate these mixtures alone or combined with in a hybrid process (for example, with distillation) improving the separation and energy efficiency [107]. The classification of organic–organic mixtures is presented in Figure 9.

The polymeric membrane can be hydrophilic or hydrophobic based on the type of separated organic–organic solution, i.e., polar–nonpolar, polar–polar, or nonpolar–nonpolar. The usage of polymeric membranes is limited due to the degradation of the performance of these membranes in organic mixtures, which causes the enlargement and loss of membrane integrity. Cross-linking or polymer mixing for membranes for the separation application is necessary based on the type of combination of mixtures being dealt with. The development of robust inorganic membranes with adjustable pore size, in which the polymer layer determines the separation mechanism, while the inorganic support provides the appropriate mechanical integrity, offers an alternative to this [297]. The physical attachment of the polymer to the inorganic substrate, followed by cross-linking, has been used in the many pervaporation membranes. The introduction of composite membranes made up of inorganic polymers has given a huge boost to successful organic–organic separation [8]. In recent years, zeolite membranes have also piqued the interest of scientists. They are favored for a variety of reasons, including customized selectivity, high flux, and low energy consumption.

Msahel et al. prepared PLA membrane using MOF as a filler. MOF is a microporous carboxylate metal-organic framework MIL-100 Fe prepared as sub-micron particles by microwave-assisted hydrothermal synthesis (Fe-MOF-MW). This has a proven stability in the air, water and organic solvents. The PLA/Fe-MOF-MW membranes were tested in the pervaporation separation of methanol/MTBE mixtures. The Fe-MOF-MW modification improved the methanol selectivity of developed membranes. The PLA membrane with 0.5 wt.% Fe-MOF-MW had the highest selectivity with a 22% increase compared to the pristine PLA membrane. Despite the amphiphilic nature of the MOF, its structure has highly hydrophilic properties, which during modification enhances the hydrophilicity of the polymer membrane. The polarity indexes of MeOH and MTBE are 0.762 and 0.124, respectively, and hence, the MeOH polarity makes its molecules more favored to permeate through the hydrophilic Fe-MOF-MW-modified membranes compared to MTBE [298].

The commercially established recovery process for bio-alcohol production is energy intensive, significantly impacting the viability of ethanol and butanol as a biofuel. Pervaporation performance of cellulose acetate propionate (CAP) and poly(1-vinylpyrrolidone-co-vinyl acetate) (PVP/PVAc) blended membranes was reported. In the work of Amarante et al. [299] pervaporation recovery of ethanol from 2-ethylhexanol was investigated using cellulose acetate propionate (CAP) and poly(1-vinylpyrrolidone-co-vinyl acetate) (PVP/PVAc) blend membranes. Cellulose-based polymers are usually hydrophilic, mainly due to the presence of acidic (OCOH) and carbonyl (CO) functional groups. Cellulose esters have been used in the synthesis of membranes for pervaporation applications, such as biofuel purification. Cellulose acetate propionate (CAP) and poly(1-vinylpyrrolidone-covinyl acetate) (PVP/PVAc) blend membranes were prepared using a solvent casting method. Fluxes and permeances of individual components, as well as separation factors and selectivity of ethanol to 2-ethylhexanol were determined at 40–60 °C, feed composition of 5–25% ethanol, and 10–30% copolymer mass fraction in the membrane. Ethanol fluxes of up to 683 g/(m^2^h) and permeances of up to 278 g/(m^2^h kPa) were observed at 50 °C. Ethanol:2-ehylhexanol selectivity of up to 5.5:1 and separation factors up to 250 were achieved, indicating that this polymeric membrane process was capable of concentrating ethanol/2-ethylhexanol solutions effectively [299].

ETBE is applied as an oxygenate additive for lessening air pollution without causing harmful impacts on human health. Moreover, ETBE can be formed from renewable sources, e.g., biomass, cellulose, bio-ethanol. Ethyl acetate (EtAc), commonly used in a broad range of applications, e.g., pharmaceuticals, petroleum, and electric industry, textile, cosmetics, is expected to witness steady growth in the future, owing to its low cost and toxicity levels. As EtAc and ETBE are industrially important organic solvents, it is beneficial to recycle both solvents from an economical and environmental point of view. Hydrophobic polymer is also suitable for this application. A fluorinated MOF-808 was designed and developed by using perfluoro carboxylic acids (i.e., trifluoroacetic acid (TFA) and pentafluoropropionic acid (PFPA)) modulators) to increase the hydrophobic character. Subsequently, modified MOF-808 (MOF808-TFA and MOF-808-PFPA) was incorporated into PDMS membrane for the pervaporation separation of organic mixtures (EtAc/ethanol, EtAc/isopropanol, and ethyl ETBE/ethanol). Separation in pervaporation takes place due to the differences in the membrane affinity for the components of the feed, whereas diffusion depends on the free volume in polymer matrix. The incorporation of MOF particles rearrange the polymer chains and consequently changes the free volume and permeation flux. Additionally, MOF particles are characterized by a porous structure. The pore sizes of MOF 808-TFA and MOF-808-PFPA are much larger than the molecular diameters of the individual compounds, which means that components can freely pass through the pores of the fillers and MOF particles serve as a highly permeable region. Therefore, those highly permeable regions created by MOF particles are available to EtAc and ETBE molecules, while ethanol and isopropanol molecules have less affinity for the MOF. As a result, the separation ability of the filled membranes was higher [200].

### 6.2. Removal of Organic Solvents from Water

Water must be removed from hydrophilic solvents before reuse, many hydrophilic solvents containing water difficult to separate because of the azeotropic mixtures. Separation procedures involving such mixtures are energy consuming and expensive. Pervaporation is a technology that has proven to be particularly effective in extracting water from azeotropic mixtures using less energy than distillation-based processes. As was already mentioned above, the solution-diffusion mechanism for the dense permselective layer of the membrane and the solvent/water mixture is the basis for the separation.

Air stripping, adsorption, advanced oxidation, distillation, anaerobic/aerobic biological treatment, bioreactor, and membrane technology are some of the treatment methods for eliminating VOCs from water [300]. There are many limitations for these methods. Because of its simplicity and low cost of operation, air stripping is commonly utilized for this purpose. It works well with high-volatile chemicals. However, it has the potential to pollute the air. Due to the high cost of adsorbents and the need for frequent regeneration, adsorption is only cost-effective at low VOC levels. Biological therapy is a safe procedure, although it takes time and is only effective at low levels of VOC. Advanced oxidation is effective for certain substances, but it can also results in the formation of new products that are more toxic than the earlier ones [300]. Hydrophobic pervaporation is the finest method for removing VOCs from water. A VOC-containing liquid stream is introduced to one side of a hydrophobic membrane, while a vacuum or sweeping gas is delivered to the other. The liquid phase’s components adhere to the membrane, permeate through it, and evaporate into the permeate side [301].

### 6.3. Organic Dehydration

Pervaporation is a technique that is effective at breaking the azeotropic mixtures of different types. Pervaporation is widely applied in chemical separation, including dehydration of organic compounds, separation of organics from water and organic mixtures. The most important industrial application of pervaporation is the dehydration of organic liquids. Pervaporation is firstly introduced in separating different types of water-organic solutions at azeotropic concentration. It is commonly used in water-acetone [302], water-isopropanol [303], water-acetic acid [304], water–butanol [305], water-ethanol, water–ethylene glycol [138], water–tetrahydrofuran [306] separations and on an industrial scale and finds its application in the solvent purification mainly for dehydration of ethanol and isopropanol. In the industrial production of ethanol, the final product is a dilute aqueous solution, and in the large scale the distillation is used to process ethanol in order to concentrate it. Since it forms an azeotrope at 96 wt.% of ethanol, the separation process is complicated. Therefore, by the convention distillation is very difficult to produce pure ethanol from azeotropic mixtures. Pervaporation is an effective alternative considering azeotropic mixtures. PAs [307], PBI [308], CS, PAN and PVA [309] are some hydrophilic polymers used as membranes for pervaporation dehydration. Among these, PVA-based membranes are used on an industrial scale [309].

Modifying the active layers of pervaporation membranes with different chemicals, these membranes exhibited much improved permeability and selectivity toward water extraction. PBI-based membranes are reported for the dehydration of various solvents such as alcohols, glycol, and acetone [287]. Wang et al. studied the performance of PBI/PEI double-layer hollow fiber membranes for ethyl acetate treatment. PBI is the outer selective layer material and PEI is the supporting layer material; effect of spinning parameters, variation of air gap distance and the take up speed on the pervaporation performance are studied in this work. The results showed that with the increase in the air-gap distance the permeance, selectivity, and separation factor all increased because of the thinner fiber wall and densified outer skin layer, while the flux decreased. Both the flux and the separation factor increased with the increase in the take-up speed. The performance of dual-layer PBI/PEI hollow fiber membrane is superior to most other developed polymeric membranes due to unique combination of the excellent physicochemical characteristic of the PBI selective layer, the less swelling attributing of the PEI supporting layer: separation factor of 2478 with a permeation flux of 820 g/(m^2^h) in the pervaporation dehydration of the 98/2 wt.% EA/water at 60 °C [310]. The discovery of graphene contributed to the development of 2D materials, which offers new opportunities for the development of new membranes with greater separation properties. Transition metal carbides (MXene) are new addition to 2D materials, which have received increasing attention in many fields including membrane separation due to good mechanical strength and conductivity. G. Liu et al. [311] reported that polyelectrolyte functionalized Ti_2_CTxMXene membrane by introducing positively charged polyelectrolytes (PAH, PEI, and PDDA) to create electrostatic attraction with the negatively charged Ti_2_CTx nanosheets for pervaporation separation of isopropanol/water mixtures. Positively charged polyelectrolytes were introduced to create electrostatic attraction with the negatively charged Ti_2_CTx nanosheets in order to achieve laminar structure of the assembled Ti_2_CTx MXene membrane. The polyelectrolytes were used to increase the water affinity of MXene membranes and to enhance the fast water transport, which is a key feature of solvent dehydration. PDDA functionalized MXene membrane possessed the highest performance for dehydration of 90 wt.% isopropanol/water at 50 °C due to electrostatic effect and hydrophilicity: a total flux of 1237 g/(m^2^h) and separation factor of 1932 [311].

TFC membranes are a promising strategy for pervaporation due to their high separation factor and high permeation flux. TFC membranes have an ultrathin PA active layer and a porous substrate providing separation function and strength. X. W. Liu et al. [312] reported TFC membranes made by interfacial polymerization (IP) of 1,3-diaminopropane (DAPE) and trimesoyl chloride (TMC) on the surface of TiO_2_ modified ceramic hollow fiber (CHF) substrate for the dehydration of isopropanol. Mesoporous TiO_2_ intermediate layer was prepared by sol gel dip coating on outer surface of porous α-Al_2_O_3_ CHF membrane, then a PA ceramic TFC was prepared using interfacial polymerization method on the porous hollow fiber substrate. Obtained results shows a permeation flux of 6.44 kg/(m^2^h) and separation factor over 12,000 for pervaporation dehydration of 90 wt.% aqueous isopropanol solution at 60 °C. Studies by Halakoo and Feng [283] showed layer by layer assembly of PEI and GO on a chemically treated TFC PA substrate for pervaporation dehydration of ethylene glycol. The hydrophilic nature of polymer in the membrane favored the selectivity towards water permeation. The coupling of PEI and GO also produced strong hydrogen bonding as well as electrostatic attraction. It was found that the alternate deposition of PEI and GO on a chlorinated TFC PA was a promising approach to assembling membranes due to electrostatic interactions and hydrogen bonds between ionized carboxyl groups in GO and protonated groups in PEI. Membranes with only three PEI/GO bilayers showed good selectivity, stability and permeation flux for dehydration of ethylene glycol [283].

Two dimensional (2D) materials with atomic thickness and micrometer lateral dimensions have been widely used to develop membranes with high separation performance. Moreover, they have mechanical properties, thermal stability, excellent layered structure, and 2D nanochannels, making the two-dimensional material separation membrane to have extraordinary permeability. Cai et al. [313] prepared 2D Ti_3_C_2_Tx and embedded it in the PVA matrix for ethanol dehydration. This material demonstrated excellent compatibility and swelling resistance. The results demonstrated good compatibility between PVA matrix and Ti_3_C_2_Tx: Ti_3_C_2_Tx was uniformly dispersed in the polymer matrix. PVA/Ti_3_C_2_Tx (3 wt.%) had the best separation performance of 93 wt.% ethanol solution at 37 °C: separation factor of 2585, which was 17 times higher than that of the pristine PVA membrane.

### 6.4. Desalination

**Figure 10:**
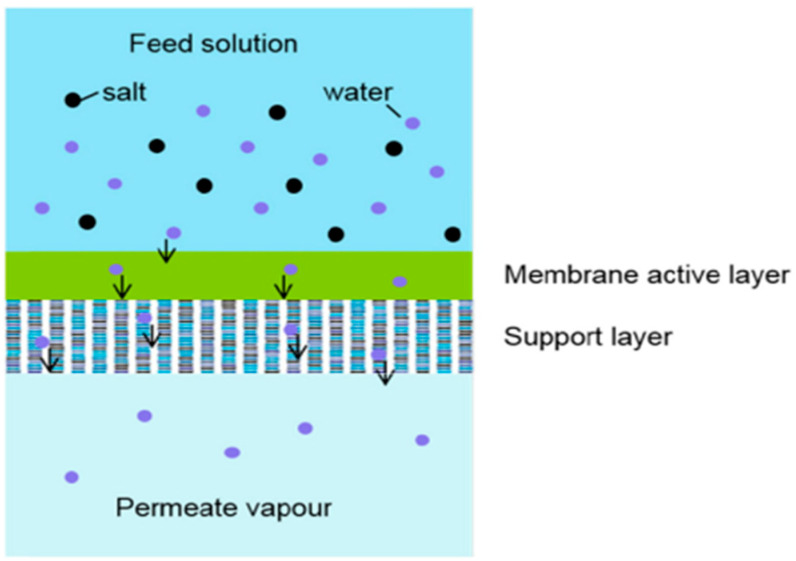
Desalination using pervaporation.

Due to the ever increasing population, the shortage of fresh water resource has become a global issue, and, to overcome these difficulties, one possible solution is to convert brakish or seawater into fresh water using desalination technologies. Currently, multi stage flash (MSF) distillation and reverse osmosis are the most commercially important technologies for the desalination, but these have many disadvantages including high power consumption and low heat transfer efficiency in the case of MSF distillation and high pressure to overcome osmotic pressure of seawater during reverse osmosis [227]. For application in desalination, pervaporation has advantages due to its 100% salt rejection, high selectivity of polymer membranes and tunable nanopores of inorganic membranes [314]. For desalination using pervaporation hydrophilic membranes are needed as they favor the transport of water molecules in the vapor state. Firstly, the water molecules are sorbed by the selective layer of membrane followed by diffusion and as a result of concentration gradient water molecules are permeated through the membrane and desorbed at the permeate site [66,315]. Pervaporation is most probably the technology offering the lowest permeation rate, but provides a high rejection of salts according to the membrane properties. The only possibility of pervaporation technology for its competition in desalination regards to the development of more water-permeable membranes. It is important to point out that pervaporation has been commercialized within hybrid purification processes for the removal of water from organics. Thereby, membranologists are still believing and extensively working in developing new membranes with enhanced separation performance for implementing them in pervaporation desalination. Most commonly used membrane technology for desalination is reverse osmosis. Even though it has many advantages, it lacks the ability to handle brine solutions of more than 4% NaCl. This can be overcome using pervaporation, where the seawater is vaporized and separated from the salt by driving the water vapor via a pressure gradient across a membrane. More recently, the research on both organic and inorganic pervaporation membranes has presented remarkable progress, which includes hybrid organic–inorganic membranes and TFC membranes with high water flux. The mechanism of desalination using pervaporation is presented in Figure 10.

TFC membranes have been identified as the high-permeable concept of membranes, e.g., PVA/PAN composite, cellulose, SiO_2_/PVA, zeolite ZSM-5, and GO/PAN composite membranes offer high permeation rates ranged from 6.7 to 65 kg/(m^2^h). Study by Meng et al. [288] showed the fabrication of a TFC membrane of photo-crosslinkable PVA-stibazol quaternised (PVA-SBQ) dense selective layer for desalinating brine solutions, containing up to 20 wt.% NaCl. PVA-SBQ was first deposited onto a nanofibrous composite porous support layer, which contained electrospun nanofiber mat coated with nanocellulose (NC) prior photo-curing with UV light (365 nm). This photo-crosslinked dense selective layer with highly porous nanofibrous support layers produced membranes with water flux of 79.9 ± 13.3 to 122.6 ± 10.8 kg/(m^2^h) and salt rejection of up to 99.9% processing brine solutions of 20 to 3.5 wt.% of NaCl.

Cha-Umpong et al. [316] found out that the deposition of the GO nanosheets on the polypropylene (PP) membranes has significantly improved Na^+^, Ca^2+^, Mg^2+^ rejection up to 99.99%. The thermal driven process showed superior water permeation and high salt rejection. The smoother and negatively charged surface from the GO nanosheets also hindered the undesired crystal deposition on the membrane surface and improved the desalination performance, and also the higher oxidation state of GO surface improved the salt rejection and the binding energy between hydrated divalent cations and oxygen functional groups. In 2020 Ding et al. [317] reported a maleic acid covalent-bridged MXene (MXMA) membrane for pervaporation desalination, vacuum-assisted filtration method was used for the fabrication of the membrane. Pervaporation desalination performance of both MXMA and MX membranes was investigated in this study. The MXMA exhibited higher water flux of 22.8 kg/(m^2^h) than that of MX membrane (17.3 kg/(m^2^h)) without the compromise of selectivity performance. Qin et al. [318] used non-solvent induced phase inversion (NIPS) method and the electrospinning technique for the fabrication of two different kinds of membranes comprising PVDF and PAN electrospun nanofiber substrates. For the deposition of thin defect free selective layer on the PAN electrospun nanofiber substrate, ultra-fine nanofiber cellulose (NC) layer was deposited on its surface by knife coating method. Due to the NC gutter layer most of the PVA molecules are rejected leading to a defect free thin PVA top layer deposited for the fabrication of TFC membrane. The TFC membrane exhibits water fluxes of 153.4 ± 3.4 kg/(m^2^h) at 70 °C and 238.7 ± 4.1 kg/(m^2^h) at 80 °C with a salt rejection over 99.8% for desalinating 3.5 wt.% NaCl solution.

## 7. Future Directions and Research Needs

In recent years, the global use of pervaporation membranes has expanded tremendously [25]. One of the fastest growing areas is the application in hybrid methods, such as combining distillation with pervaporation [319,320], which have a lot of promise, especially when high product purity is required. Hybrid systems, which comprise of two or more separation procedures of different types in sequence, are used to decrease expenses, energy costs, make a difficult separation achievable, and enhance the degree of separation [27]. Hybrid systems such as distillation with pervaporation minimize energy consumption, enable separations that would otherwise be impossible, and enhance the degree of separation. Despite the relevance of the use of pervaporation both separately and in hybrid processes, its wide industrial application has not yet been achieved due to its high sensitivity to operating conditions, the lack of specialized efficient and stable membranes, and the high cost of these membranes [27].

The environmental impact of plastic wastes is a global problem, and recycling technologies are limited [321]. Existing law aimed at environmentally friendly and sustainable technology is increasingly pushing for newer alternatives and more competitive methods that meet economic and environmental norms. Pervaporation is undoubtedly at the forefront of this framework as an energy saving and eco-friendly, efficient and appealing technology capable of competing with classic and well-established technologies such as distillation, liquid-liquid extraction, and adsorption. However, pervaporation has still a low sustainability due to membrane fabrication from fossil-based polymers, which mostly have a high cost. The growing pollution of the environment has become a starting point for research on potential natural polymers that can replace conventional polymers for membrane preparation [322]. Biopolymers obtained from animals (polylactic acid, polyhydroxyalkanoates, polybutylene succinate), plant sources (cellulose-based polymers, alginate, polyisoprene, starch), bacterial fermentation products (chitin, chitosan, collagen, sericin), and biodegradable polymers (polyvinyl alcohol, polylactic acid, etc.) have attracted the attention of researchers throughout for many years with a growing global trend towards sustainable development [322]. The main obstacles to the development, scaling and market entry of biopolymer membranes are problems with their solubility and mechanical strength [323]. In addition, there are limited reports of upcycling and recycling biopolymer membranes after use to determine resistance [323]. Researchers still have to solve the problems of biopolymers for maximum and industrial application in membrane technologies. Moreover, to make pervaporation more sustainable except for biopolymer application, other strategies can be applied during membrane fabrications: (1) the use of environmentally friendly “green” solvents (dimethyl sulfoxide (DMSO), methyl lactate, ethyl lactate, PolarClean, Cyrene, TamiSolve NxG, N,N-dimethyl lactamide and ionic liquids and etc.) [324]; (2) replacement of traditionally used toxic organic solvents for membranes by non-toxic and environmentally friendly synthetic organic solvents, solvents based on renewable raw materials and ionic liquids [325]; (3) replacement of the monomers and functional additives synthesized from petroleum, which are widely used in membrane production, by non-toxic, environmentally friendly monomers and additives based on renewable raw materials [326,327].

## 8. Conclusions

In this review, mass transport mechanisms within pervaporation membranes, material selection for pervaporation membranes (polymeric membranes, inorganic membranes, membranes based on 2D materials, mixed matrix membranes), fabrication techniques (the physical blending method, hollow fiber spinning, in-situ polymerization, layer-by-layer assembly method, sol-gel method, bioinspired methods, photo-crosslinking, solid solution casting and solution coating methods), and pervaporation membrane characterization methods are presented.

**Polymers** are the largest family of membrane materials for pervaporation due to their low cost and ease of preparation. Depending on the affinity, hydrophilic polymers are used to develop membranes for selective permeation of water over organics, and hydrophobic polymers—for organics. There is a large number of hydrophilic polymers for the preparation of pervaporation membranes such as PVA, PEC, CS, SA, cellulose derivatives, PA, PI, and so forth. Among this, PVA is one of the first commercialized pervaporation membrane materials, which remains as the benchmark polymer of hydrophilic membranes for solvent dehydration. Despite the low stability of thin selective layers based on polyelectrolytes or PA obtained by interfacial polymerization, they are very promising due to the resulting tailored transport properties of the membranes. However, it should be mentioned that, due to the focus of society on sustainable processes, nowadays, biopolymers are the most used alternative to chemically synthesized polymers in the manufacture of membrane materials. Among them, CS, SA, and cellulose derivatives are widely applied in the production of pervaporation membranes. Among hydrophobic polymers for the preparation of pervaporation membranes, PDMS is the most widely used membrane material exhibiting hydrophobicity, processability and stability in addition to excellent separation performance. Other polymers that are widely used are PTMSP, PVC, and PIM-1.

**Inorganic membranes** with well-defined and rigid pore structures exhibit great separation performance and stability in pervaporation process. They are generally prepared from crystalline microporous materials such as zeolite, COF, and MOF. Like polymers, inorganic membranes are also classified into hydrophobic and hydrophilic. Zeolite membranes are the most intensively studied, while the development of crystalline membranes from MOF and COF for pervaporation is still at an early stage.

**Two-dimensional (2D) materials** are a new family of membrane materials since the discovery of graphene, which has received much attention (graphene, graphene oxide (GO), MXenes, 2D transition metal carbides and/or nitrides, layered transition metal dichalcogenides, layered zeolites, 2D MOFs and 2D COFs). It is expected a significant reduction in membrane thickness because of the atomic thickness of 2D materials causing fast component transport through the pores or interlayer channels of the membrane (increased permeability and selectivity of separation). Currently, pervaporation membranes from 2D material mainly used for dehydration of organic matter. Among 2D materials for membrane separation, graphene-based materials has its own prior position as it has the advantages in single-carbon-atom thin and large lateral size.

In addition to the pervaporation membranes from a single material type, **mixed matrix membranes (MMMs)** are prepared by introduction of various fillers into a polymer matrices and have attracted a tremendous attention since the 1990s. This approach allows solving the trade-off problem of permeability-selectivity by proper selection of optimal modifiers, which may significantly change structural and physicochemical characteristics (surface functionalization, change of free volume, morphology, etc.) causing improved transport properties. Depending on the separation task, hydrophobic or hydrophilic fillers may be used to prepare MMMs enhancing the membrane adsorption towards water or organics.

The application of pervaporation membranes was demonstrated in this review for organic–organic separation, the removal of organic solvents from water, organic dehydration and desalination. Pervaporation’s ability to separate azeotropes, close-boiling mixtures and isomers is one of the key benefits that makes it more attractive and efficient than other traditional procedures such as distillation with the right choice of membrane material. For treating VOC-contaminated water, this approach is both technically and economically practical. The method is concise, continuous, and does not employ materials that are easily depleted. The selective membrane layer can be made of a range of polymers, allowing for the flexibility needed to remove certain organic contaminants. Pervaporation, unlike any other common technology, allows for the direct recovery of organic molecules for reuse in industrial operations.

In terms of membrane manufacture as well as the use of this approach in a day-to-day setting, there is still a long way to go. Moreover, for the effective pervaporation application, the development of a platform for the fabrication of advanced and novel polymer materials including more sustainable membrane-enabled separation technologies is needed to increase the separation performance and effectiveness of industrially significant liquid media. It should be noticed that to present a complete in-depth review of all aspects of pervaporation separation is impossible as in this membrane process many aspects are still under study and not understood. This review attempted to present some important aspects in this quickly developing field, which needed to be solved or clarified.

## Data Availability

Not applicable.
